# Selective photoinduced charge separation in perylenediimide-pillar[5]arene rotaxanes

**DOI:** 10.1038/s41467-022-28022-3

**Published:** 2022-01-20

**Authors:** Nicholas Pearce, Katherine E. A. Reynolds, Surajit Kayal, Xue Z. Sun, E. Stephen Davies, Ferdinando Malagreca, Christian J. Schürmann, Sho Ito, Akihito Yamano, Stephen P. Argent, Michael W. George, Neil R. Champness

**Affiliations:** 1 School of Chemistry, University of Birmingham, Edgbaston, Birmingham B15 2TT UK; 2 School of Chemistry, University of Nottingham, University Park, Nottingham NG7 2RD UK; 3 Rigaku Europe SE, Hugenottenallee 167, 63263 Neu-Isenburg, Germany; 4 Rigaku Corporation, 3-9-12, Matsubara, Akishima, Tokyo 196-8666 Japan

**Keywords:** Interlocked molecules, Self-assembly, Photochemistry, Light harvesting

## Abstract

The ability to control photoinduced charge transfer within molecules represents a major challenge requiring precise control of the relative positioning and orientation of donor and acceptor groups. Here we show that such photoinduced charge transfer processes within homo- and hetero-rotaxanes can be controlled through organisation of the components of the mechanically interlocked molecules, introducing alternative pathways for electron donation. Specifically, studies of two rotaxanes are described: a homo[3]rotaxane, built from a perylenediimide diimidazolium rod that threads two pillar[5]arene macrocycles, and a hetero[4]rotaxane in which an additional bis(1,5-naphtho)-38-crown-10 (BN38C10) macrocycle encircles the central perylenediimide. The two rotaxanes are characterised by a combination of techniques including electron diffraction crystallography in the case of the hetero[4]rotaxane. Cyclic voltammetry, spectroelectrochemistry, and EPR spectroscopy are employed to establish the behaviour of the redox states of both rotaxanes and these data are used to inform photophysical studies using time-resolved infra-red (TRIR) and transient absorption (TA) spectroscopies. The latter studies illustrate the formation of a symmetry-breaking charge-separated state in the case of the homo[3]rotaxane in which charge transfer between the pillar[5]arene and perylenediimide is observed involving only one of the two macrocyclic components. In the case of the hetero[4]rotaxane charge separation is observed involving only the BN38C10 macrocycle and the perylenediimide leaving the pillar[5]arene components unperturbed.

## Abstract (web summary)

The development of complex molecular machinery requires a detailed appreciation of the factors that control energy pathways through the nanoscale scaffold. Here, the authors demonstrate that hetero-rotaxanes can be employed to create assemblies of different redox and photo-active components that enable selective tuning of energy transfer pathways.

## Introduction

The development of complex molecular machinery and functional molecular assemblies requires a detailed appreciation of the factors that control energy pathways through the nanoscale scaffold^1–7^. Understanding and directing energy transfer pathways represents a challenge both in terms of synthetic manipulation of molecules but also in terms of detailed characterisation of the interplay between the components of the assembled system. One approach to developing our understanding in this avenue of research is to create assemblies composed of molecular components that exhibit redox activity and/or optical properties that can be studied by a combination of spectroscopic and electrochemical techniques. In this regard, supramolecular assemblies based on redox-active molecules have been developed, notably for their potential in photosynthetic light harvesting^2,8–11^ and in organic electronics^12–14^. In turn, molecular dyads that comprise both electron-accepting and electron-donating groups are attractive for these purposes, potentially allowing for energy conversion following a photochemical reaction^5,15,16^. Much effort in this field has focussed on the development of covalently linked systems establishing an appreciation of the effects of donor–acceptor spatial separation^5,16–18^. However, synthetic approaches to mechanically interlocked molecules (MIMs)^19^ and molecular nanotopology^20^ allow the creation of increasingly complex systems with controlled spatial separation of electrochemically and optically active centres^3,4,7,21–24^. For example, elegant research by the groups of Guldi and Schuster^4^ has led to the creation of multichromophoric mechanically-linked charge transfer cascade systems with charge-separated lifetimes reaching into the microseconds.

In this study, we show that hetero-rotaxanes can be employed to create assemblies of different redox- and photo-active components that enable selective tuning of energy transfer pathways. In particular, we have studied rotaxanes formed using a rod composed of a redox- and photo-active perylenediimide (PDI) functionalised with two imidazolium arms. This rod presents two chemically distinct recognition sites for rotaxane formation; the PDI and the two imidazolium groups. The imidazolium groups are effective recognition sites for electron-rich pillar[5]arene macrocycles, where size and charge complementarity between these two groups lead to an efficient host–guest interaction. Pillar[5]arenes, whose cavity is too small to encompass the PDI, have recently been demonstrated to form a stable radical upon reversible oxidation in anhydrous CH_2_Cl_2_, even when functioning as a host molecule to alkylimidazolium guests^25^. The PDI itself presents an auxiliary recognition site and is suited to forming host–guest complexes with a bis(1,5-naphtho)-38-crown-10 (BN38C10) macrocycle^26^ which is known to act as an electron-donating species^27^.

**Fig. 1:**
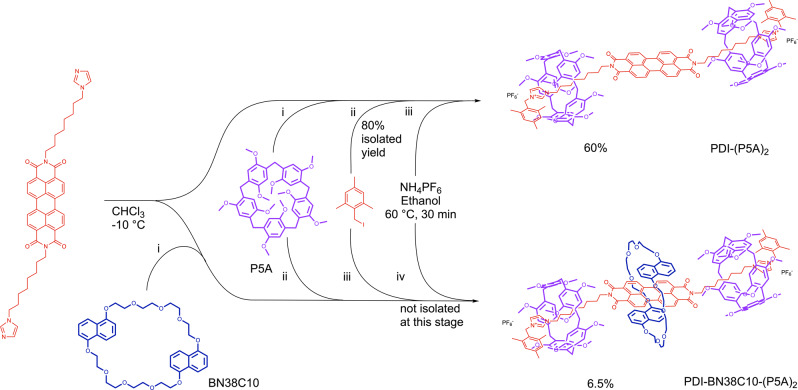
Synthesis of PDI-rotaxanes. Upper pathway: [3]rotaxane, [PDI-(P5A)_2_](PF_6_)_2_, and lower pathway: [4]rotaxane, [PDI-BN38C10-(P5A)_2_](PF_6_)_2_. The PDI bis-imidazolium axle, and its constituent parts, are shown in red; pillar[5]arene in purple and BN38C10 in blue.

Our strategy enables the study of both a homo[3]rotaxane, [PDI-(P5A)_2_](PF_6_)_2_, and a hetero[4]rotaxane, [PDI-BN38C10-(P5A)_2_](PF_6_)_2_ (Fig. 1). Although each rotaxane contains only one photo-active electron-accepting component, the PDI, the two rotaxanes differ in terms of the potential donor macrocycles. Whereas [3]rotaxane [PDI-(P5A)_2_](PF_6_)_2_ contains only one type of donor, the P5A, [4]rotaxane [PDI-BN38C10-(P5A)_2_](PF_6_)_2_ contains two distinct donors, P5A and BN38C10, presenting two distinct pathways for energy transfer processes. Using time-resolved infra-red (TRIR) spectroscopy we show that photoinduced charge transfer in hetero[4]rotaxane [PDI-BN38C10-(P5A)_2_](PF_6_)_2_ proceeds between the PDI and BN38C10 components and does not involve pillar[5]arene groups, as observed in [PDI-(P5A)_2_](PF_6_)_2_, thus the pathway of energy transfer in these mechanically interlocked rotaxanes is directed by the nature and relative organisation of the macrocycles.

## Results and discussion

### Molecular design and synthesis

The typical strategy for the synthesis of pillararene-based rotaxanes relies upon the strong interaction between the electron-rich cavity of P5A and electron-deficient threading groups, such as imidazoles^25,28,29^. When mixed in chloroform solution, a pseudorotaxane inclusion complex is formed, which can be reacted with appropriately bulky caps to prevent dissociation of the molecular components. Thus, we synthesised an *N*,*N*′-symmetrical PDI equipped with octylimidazole groups to serve as the axle of the two different rotaxanes, providing the necessary threading groups for interactions with the P5A macrocycles. A symmetrical [3]rotaxane, [PDI-(P5A)_2_](PF_6_)_2_, was synthesised by dissolving the PDI axle in a minimum quantity of CHCl_3_ followed by the addition of an excess of P5A whilst sonicating for 10 min to promote threading, before cooling in a salt-ice bath and adding excess 2,4,6-trimethylbenzyl iodide to cap the ends of the axle via alkylation of the free nitrogen atom on the imidazole (Fig. 1). [PDI-(P5A)_2_](I)_2_ was produced in high yield (80%) and, to allow for electrochemical characterisation, the iodide counter ions that originate from the stopper group were exchanged for PF_6_^−^ to afford [PDI-(P5A)_2_](PF_6_)_2_.

We have previously demonstrated that the bis-naphtho-crown ether macrocycle, BN38C10, interacts well with PDIs through π–π stacking, and can be used to construct PDI-containing rotaxanes^26^, and so we reasoned that a homologue of this [3]rotaxane could be created in which the central PDI is encapsulated within the cavity of BN38C10, which is in turn trapped by the large flanking P5A macrocycles. An adapted synthetic strategy was employed for the synthesis of this hetero[4]rotaxane whereby the PDI bis-imidazole was initially mixed with a stoichiometric equivalent of BN38C10, leading to a colour change of the solution from red to purple (illustrating the complexation of the red PDI by BN38C10), before repeating the sequential pillararene-threading, end-capping and anion exchange procedures (Fig. 1). The [4]rotaxane, [PDI-BN38C10-(P5A)_2_](PF_6_)_2_, was initially purified with silica gel flash chromatography, however, isolation of the pure [4]rotaxane from other reaction products was more challenging. The most prevalent side-product of the reaction, perhaps unsurprisingly, was found to be the [3]rotaxane, [PDI-(P5A)_2_](PF_6_)_2_, most likely from reaction of PDI that had not associated with BN38C10 prior to the addition of the pillararene. Separation of the [3]- and [4]rotaxanes was not achieved using chromatography techniques, but instead the [4]rotaxane could be selectively crystallised by layering MeOH/hexane onto a CHCl_3_ solution of the mixture, regularly removing the purple crystals of pure [4]rotaxane soon after they began to form, and repeating the crystallisation as necessary until the solution colour turned from purple to red and no further crystals could be obtained.

Characterisation of the rotaxanes was possible through a combination of MALDI-TOF mass spectrometry and NMR spectroscopy. The MALDI-TOF spectrum showed strong signals for the rotaxanes due to the cationic axles, observed primarily as a singly charged species, a mono-cation with accompanying PF_6_^−^ ion. The ^1^H NMR spectrum confirmed the presence of a threaded species through upfield shifts of the aliphatic chain on the PDI, diagnostic for pillar[5]arene-rotaxane formation^28,29^. The P5A aromatic protons were also split into two chemically distinct environments by the threading reaction, due to the asymmetry of the axle: the ^1^H environments of the rim of the pillararene facing the stopper are shifted downfield relative to those of the rim facing the PDI. In the ^1^H NMR of the [4]rotaxane, the PDI signals are shifted upfield to a singlet at 8.51 ppm, when compared with the expected two doublets of *N*,*N*′-symmetrically substituted PDIs, as seen for the [3]rotaxane. This shift and splitting pattern are identical to that observed for our earlier PDI-BN38C10 [2]rotaxane^26^. The relatively simple ^1^H and ^13^C NMR spectra for both the rotaxanes indicate the underlying symmetry of the molecules: both pairs of pillararene units and their aliphatic axles are equivalent. Furthermore, only 4 signals are observed for the glycolic loops of the crown ether in the ^13^C NMR spectrum of the [4]rotaxane, consistent with local C_2h_ symmetry of the macrocycle.

**Fig. 2:**
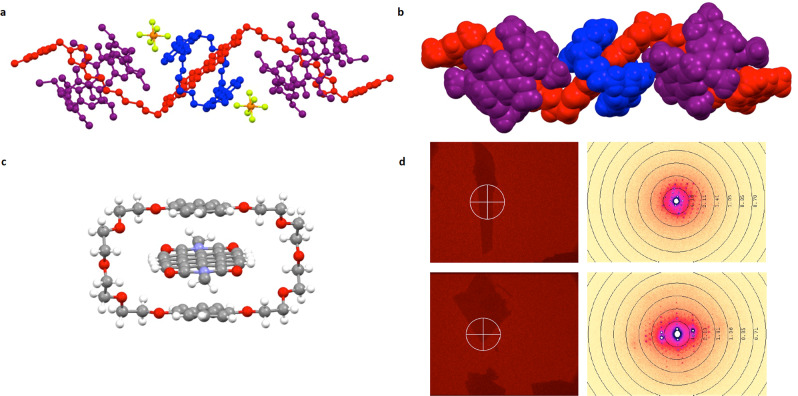
Crystallographic studies of [PDI-BN38C10-(P5A)_2_](PF_6_)_2_. **a** View of the crystal structure of [PDI-BN38C10-(P5A)_2_](PF_6_)_2_ determined using electron diffraction. The cationic PDI-imidazolium axle is shown in red, P5A macrocycles in purple, BN38C10 in blue, PF_6_^−^ anions are also shown but hydrogen atoms are omitted for clarity; **b** a space filling representation of the [PDI-BN38C10-(P5A)_2_]^2+^ rotaxane, PF_6_^−^ anions are removed for clarity and the same colour scheme is used as in (**a**); **c** π–π stacking interactions are evident between the naphthyl groups of BN38C10 and the central PDI group; **d** examples of electron diffraction patterns alongside grain snapshots of the [PDI-BN38C10-(P5A)_2_](PF_6_)_2_ crystallites.

It was possible to grow single crystals of the [4]rotaxane by layering of a 1:1 hexane:MeOH mixture onto a CHCl_3_ solution of the compound which permitted characterisation by X-ray crystallography. The [4]rotaxane, [PDI-BN38C10-(P5A)_2_](PF_6_)_2_, crystallised in the triclinic space group *P*-1, with one half of the molecule in the asymmetric unit, such that the solid-state structure mirrors some of the symmetry observed in solution (Fig. 2). Although the crystals were poorly diffracting, even using synchrotron radiation, it was clear that the naphthyl groups of BN38C10 form π–π stacking interactions with the PDI. The P5A macrocycles are disordered due to their planar chirality, with both enantiomers present in equal quantities in the structure, but their presence confirms the target structure. The refinement was highly challenging, requiring a large number of restraints and for some atoms to be placed in calculated positions prior to refinement. In addition, we were unable to locate or even identify anions in the X-ray structure. The poor quality of the refinement from X-ray data led us to explore electron diffraction as an alternative method of acquiring structural data. Thus, electron diffraction data were collected on ten separate crystallites, with approximately 100 nm thickness at room temperature (see Fig. 2 and Supplementary Information for details). The data merged across nine of these data sets, allowed structure determination giving rise to a higher quality refinement which even allowed identification and refinement of the anions. The resulting structure confirms the overall arrangement of [PDI-BN38C10-(P5A)_2_](PF_6_)_2_ and verifies the adoption of π–π interactions between the PDI and the naphthyl groups of the BN38C10 macrocycle. The close-proximity, 3.49 Å shortest interplanar separation, of these groups is significant in the context of the electrochemical and photophysical behaviour, see below. To our knowledge, this is the first example of electron diffraction being used to determine the structure of a mechanically interlocked molecule^30–32^. We also believe this is the first crystal structure determined for a heterorotaxane by either X-ray or electron diffraction.

### Rotaxane ground state properties

**Fig. 3:**
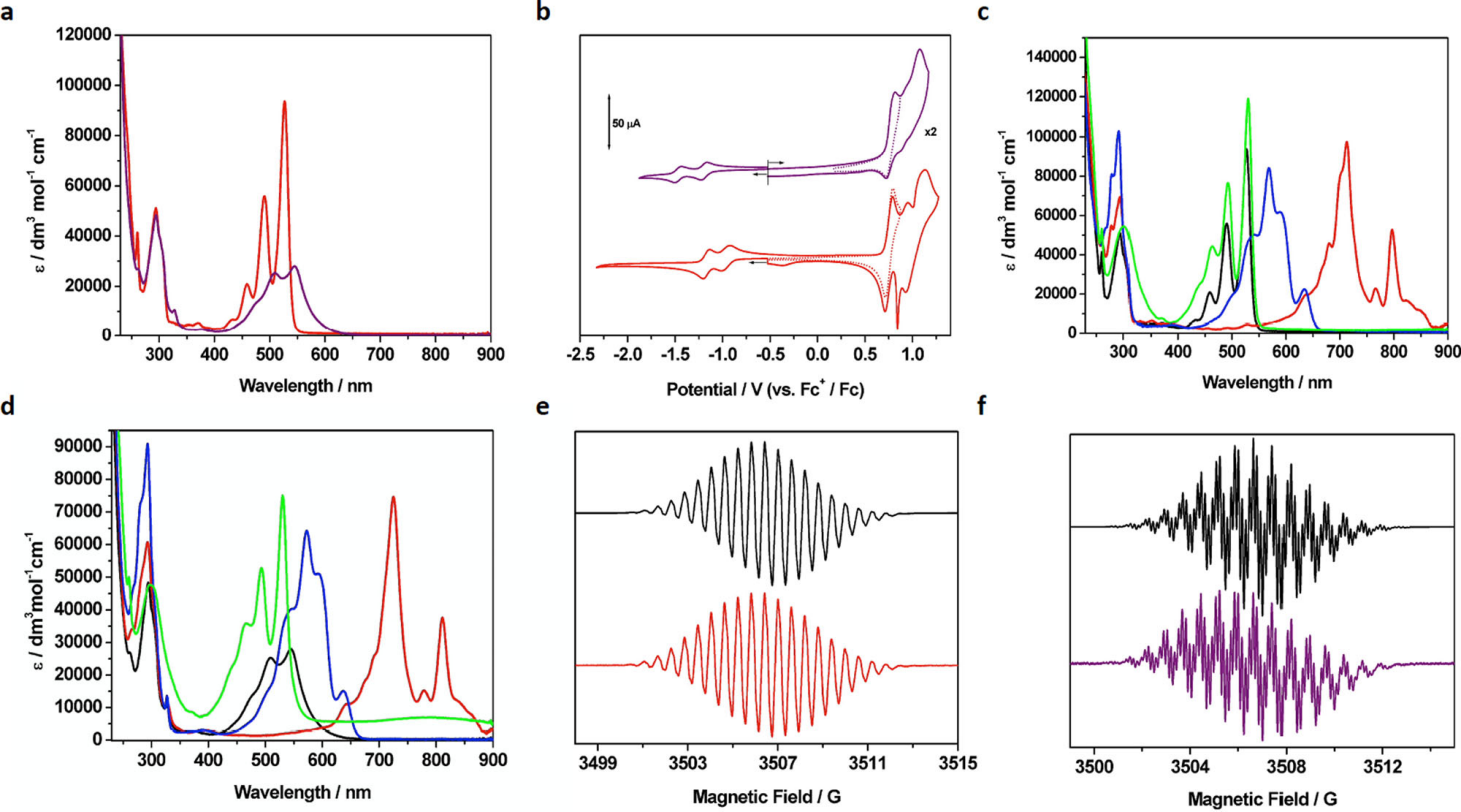
Electrochemical and spectroelectrochemical studies of [PDI-(P5A)_2_]^2+^ and [PDI-BN38C10-(P5A)_2_]^2+^. **a** UV/vis absorbance spectra of [PDI-(P5A)_2_]^2+^ (red) and [PDI-BN38C10-(P5A)_2_]^2+^ (purple); **b** cyclic voltammograms of [PDI-(P5A)_2_]^2+^ (red) and [PDI-BN38C10-(P5A)_2_]^2+^ (purple); **c** comparison of UV/vis absorbance spectra of the [PDI-(P5A)_2_]^2+^ in various oxidation states: [PDI-(P5A)_2_]^2+^ - black; [PDI-(P5A)_2_]^+^ - red; [PDI-(P5A)_2_] - blue; [PDI-(P5A)_2_]^*x*+^ - green; **d** comparison of UV/vis absorbance spectra of the [PDI-BN38C10-(P5A)_2_]^2+^ in various oxidation states: [PDI-BN38C10-(P5A)_2_]^2+^ - black; [PDI-BN38C10-(P5A)_2_]^+^ - red; [PDI-BN38C10-(P5A)_2_] - blue; [PDI-BN38C10-(P5A)_2_]^*x*+^ - green; **e** EPR spectrum of radical species formed from one electron reduction of [PDI-(P5A)_2_]^2+^ (experimental - red; simulated - black); **f** EPR spectrum of radical species formed from one electron reduction of [PDI-BN38C10-(P5A)_2_]^2+^ (experimental - purple; simulated - black). UV-visible spectra and cyclic voltammograms were recorded in CH_2_Cl_2_ containing [^n^Bu_4_N][BF_4_] (0.4 M) as the supporting electrolyte at 273 K and room temperature respectively.

Typical for most PDI compounds, [PDI-(P5A)_2_]^2+^ forms intense red solutions in chlorinated solvents, though the usual strong fluorescence is diminished substantially. The ground state absorption spectrum of [PDI-(P5A)_2_]^2+^ can be described as a combination of the absorption spectra of its constituent parts. Primarily, an intense absorbance is measured between 550 and 420 nm that is attributed to the PDI S_0_ to S_1_ transition, displaying vibronic progression^33^. An additional peak at 294 nm (*ε* = 51000 M^−1^cm^−1^) is attributed to the two P5A units^25^ (Fig. 3a). The absorbance profile of the [4]rotaxane is redshifted slightly and the overall intensity of the S_0_ to S_1_ transition is diminished in comparison to the corresponding [3]rotaxane, alongside a change in the intensity ratios of the vibronic bands: the 0 → 0 band is weaker and the 0 → 1 band is stronger than observed for the [3]rotaxane (Fig. 3a). These changes are consistent with spectral changes caused by π−π stacking with the naphthalene units of the crown ether^34,35^, as was reported previously for the neutral-state spectrum of the PDI-BN38C10 [2]rotaxane^26^. The electrochemical properties of the rotaxanes were investigated by cyclic voltammetry in anhydrous CH_2_Cl_2_ (Fig. 3b). Much like the absorption spectrum, the electrochemical behaviour of [PDI-(P5A)_2_]^2+^ can be explained by consideration of its redox active components. At negative potentials, two reversible one-electron reduction waves are observed at −0.96 and −1.18 V (vs. Fc^+^/Fc), typical for PDIs bearing no core substituents and comparable to that of the free axle^29^; whilst at positive potentials, one reversible couple was accompanied by a series of oxidation processes at higher potentials, resembling the behaviour observed for a simple pillararene-rotaxane^25^, with the first process occurring at 0.75 V (vs. Fc^+^/Fc). UV/vis absorbance spectroelectrochemical methods were used to establish the effect of these redox processes on the absorbance spectrum of the rotaxane. During the reduction of [PDI-(P5A)_2_]^2+^, the S_0_ to S_1_ absorbance bands between 550 and 420 nm are diminished and replaced with a broad band of similar intensity at 713 nm, turning the solution a deep blue colour as expected for PDI anions^29^. A slight increase in absorbance below 300 nm is also observed, overlaying the pillar[5]arene profile. The second reduction also produces the expected change, with the primary absorbance band for the PDI anion at 713 nm becoming replaced with one at 568 nm. Both reductions were reversible, and the original spectral profile of [PDI-(P5A)_2_]^2+^ was restored upon reoxidation. Following oxidation of the sample, the characteristic PDI absorbance bands increased in intensity accompanied by the peak at 294 nm broadening. Oxidation of pillararene causes an increase in absorbance in the 400–500 nm range^25^, so the increase in intensity of the PDI absorbance is most likely due to the coincidence in absorbance bands. EPR spectroscopic studies of the monoreduced and oxidised forms of [PDI-(P5A)_2_]^2+^ agree with UV/vis based assignments; analysis is consistent with the unpaired electron interacting with PDI localised orbitals upon reduction^29^ (Fig. 3c) and a pillararene-based orbital upon oxidation^25^.

In comparison to [PDI-(P5A)_2_]^2+^, the reduction potentials of [PDI-BN38C10-(P5A)_2_]^2+^ occur at more negative potentials, −1.19 and −1.47 V (vs. Fc^+^/Fc), representing a cathodic shift of over 200 mV. Interactions with the crown ether increases the electron density around the PDI chromophore, decreasing its electron affinity; indeed, the first reduction of [PDI-BN38C10-(P5A)_2_]^2+^, occurs at almost the same potential as the second reduction of [PDI-(P5A)_2_]^2+^. Although the previously reported PDI-BN38C10 [2]rotaxane exhibited a singular one electron reduction process, also at −1.19 V (vs. Fc^+^/Fc)^26^, the second reduction process was not observed, rather bulk reduction and reoxidation led to dethreading of the rotaxane. In the case of [PDI-BN38C10-(P5A)_2_]^2+^, no dethreading occurred following reduction, and both one-electron processes were found to be fully reversible. Additionally, a reversible oxidation occurs at +0.76 V (vs. Fc^+^/Fc), which was ascribed to the loss of electrons from the P5A units of the [4]rotaxane, followed by a broad, quasi-reversible wave representing loss of further electrons from the macrocycles. Overlap of oxidation waves from P5A and BN38C10 species meant that individual oxidation processes could not be resolved.

UV/vis spectroelectrochemical measurements gave further insight into both the absorption profiles of the two species but also the stability of reduced and oxidised species. Thus, spectroelectrochemical experiments generating the mono- and di-reduced species of [PDI-BN38C10-(P5A)_2_]^2+^ gave spectral profiles with features identical to those found in the corresponding reduced states of [PDI-(P5A)_2_]^2+^ albeit a small redshift in the spectra for mono- and di-reduced [PDI-BN38C10-(P5A)_2_]^2+^ is noted, by 10 and 4 nm, respectively. The similarity of the reduced state spectra suggests that following reduction the BN38C10 molecule is no longer interacting strongly with the PDI core, perhaps due to electrostatic repulsion between the reduced PDI core and the electron rich alkoxynaphthyl groups. Indeed, previous electrochemical characterisation of catenanes constructed from BN38C10 and aromatic diimide macrocycles indicate a translational rearrangement of the macrocycle occurs following a one-electron reduction process that eliminates donor–acceptor complex formation^35^. The EPR spectrum of the radical formed by single reduction of [PDI-BN38C10-(P5A)_2_]^2+^ (Fig. 3f) reveals better resolved hyperfine coupling than that of corresponding radical formed from [PDI-(P5A)_2_]^2+^, although the spectrum could be simulated well using the same number of coupling parameters: three sets of four equivalent protons and a pair of equivalent nitrogen atoms (representing the 4 *bay* and 4 *ortho* protons of the PDI core, along with its 2 nitrogen atoms and the first pair of methylene protons on the attached carbon atom). The presence of the BN38C10 macrocycle may influence the magnitude of the coupling constants, but no further coupling between the PDI radical and macrocycle was identified. Oxidation of [PDI-BN38C10-(P5A)_2_]^2+^ during spectroelectrochemical analysis produced a steady-state absorbance spectrum that also corresponded well with that of the [3]rotaxane analogue, displaying the same relative intensities of the vibronic peaks. However, returning to the neutral state did not regenerate the initial spectrum of parent [PDI-BN38C10-(P5A)_2_]^2+^, instead affording a spectrum almost indistinguishable from that of the [PDI-(P5A)_2_]^2+^ and preserving the vibronic structure. We reason that upon bulk electrochemical oxidation, [PDI-BN38C10-(P5A)_2_]^2+^ loses the additional BN38C10 macrocycle, decomposing to the bis-pillararene [3]rotaxane, [PDI-(P5A)_2_]^2+^; an observation confirmed by subsequent MALDI-TOF mass spectrometry of material recovered from the electrolysis experiment. This is an interesting contrast to the dethreading observed upon reduction of the previously reported PDI-BN38C10 rotaxane^26^. Dethreading of the BN38C10 macrocycle, presumably driven by electrostatic repulsion between positively charged macrocyles, can only be achieved by the BN38C10 macrocycle passing over one of the P5A components. Inspection of the dimensions of the two macrocycles suggests that BN38C10 does indeed have an internal area that is large enough to pass over P5A and considering the additional flexibility of the crown ether, in comparison to the comparatively rigid nature of P5A, we propose that this process provides a pathway for dethreading. Indeed, ring-through-ring threading has been reported previously for rotaxanes comprising of crown ether macrocycles^36^.

**Fig. 4:**
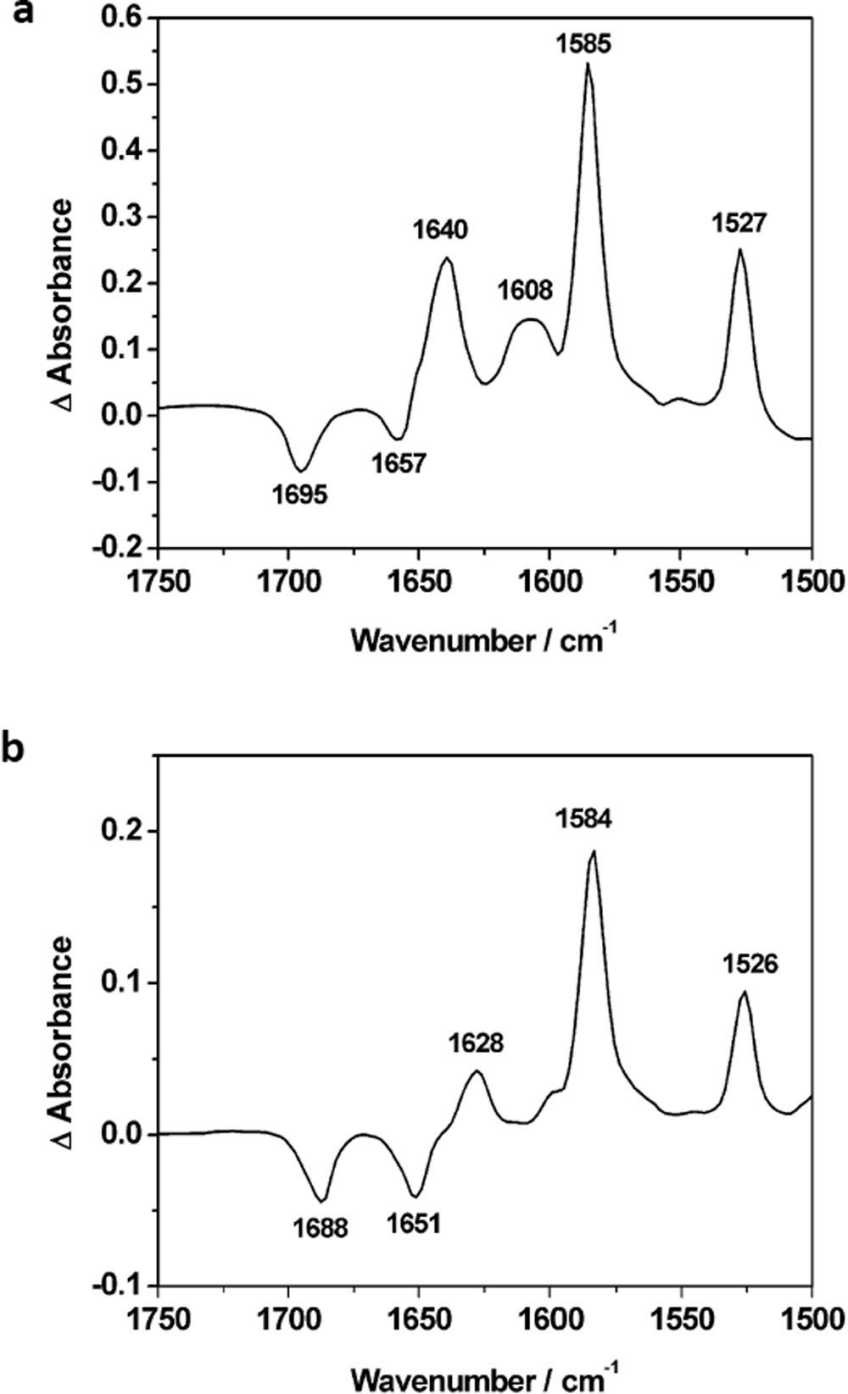
IR studies of [PDI-(P5A)_2_]^2+^ and [PDI-BN38C10-(P5A)_2_]^2+^. FT-IR difference spectra of **a** [PDI-(P5A)_2_]^2+^ and **b** [PDI-BN38C10-(P5A)_2_]^2+^ showing the change in the IR spectrum following reduction to [PDI-(P5A)_2_]^+^ or [PDI-BN38C10-(P5A)_2_]^+^ respectively. Bands with a negative change in absorbance represent bleaching of the parent rotaxane species, bands with a positive change in absorbance represent the new carbonyl stretches in the mono-reduced rotaxanes. All spectra recorded as a solution in CH_2_Cl_2_ containing [^n^Bu_4_N][BF_4_] (0.4 M) as the supporting electrolyte, at ambient temperature.

Changes in the UV/vis absorbance spectra were accompanied by related changes in the IR spectra of the monoreduced species of both rotaxanes, [PDI-(P5A)_2_]^2+^ and [PDI-BN38C10-(P5A)_2_]^2+^ (Fig. 3). Following a single reduction process to afford [PDI-(P5A)_2_]^+^ or [PDI-BN38C10-(P5A)_2_]^+^, the imide carbonyl stretches are observed at lower frequency: 1640, 1585 and 1527 cm^−1^ for [PDI-(P5A)_2_]^+^; 1628, 1584 and 1526 cm^−1^ for [PDI-BN38C10-(P5A)_2_]^+^. This shift in carbonyl frequency is consistent with the reduction of the PDI moiety in each rotaxane^37^. Despite the retention of small quantities of the parent species, the difference spectrum (Fig. 4) clearly indicates the growth of distinct carbonyl stretches for the reduced rotaxanes. The different behaviour observed for the two rotaxanes, notably in the highest frequency band (1640 vs 1628 cm^−1^) is consistent with the PDI sitting in distinct environments. These IR spectra of the reduced rotaxanes also afford a fingerprint for monitoring electron transfer processes associated with the photoactive PDI group and, therefore, allow further time-resolved characterisation of the photophysical processes of the rotaxanes.

### Rotaxane excited state properties

Having established the electron-donating and accepting properties of the PDI-rotaxanes, the photophysical properties of these compounds were investigated using time-resolved infrared (TRIR) and transient absorption (TA) spectroscopy. Both rotaxanes were probed after selective photoexcitation of PDI at 532 nm in CH_2_Cl_2_.

**Fig. 5:**
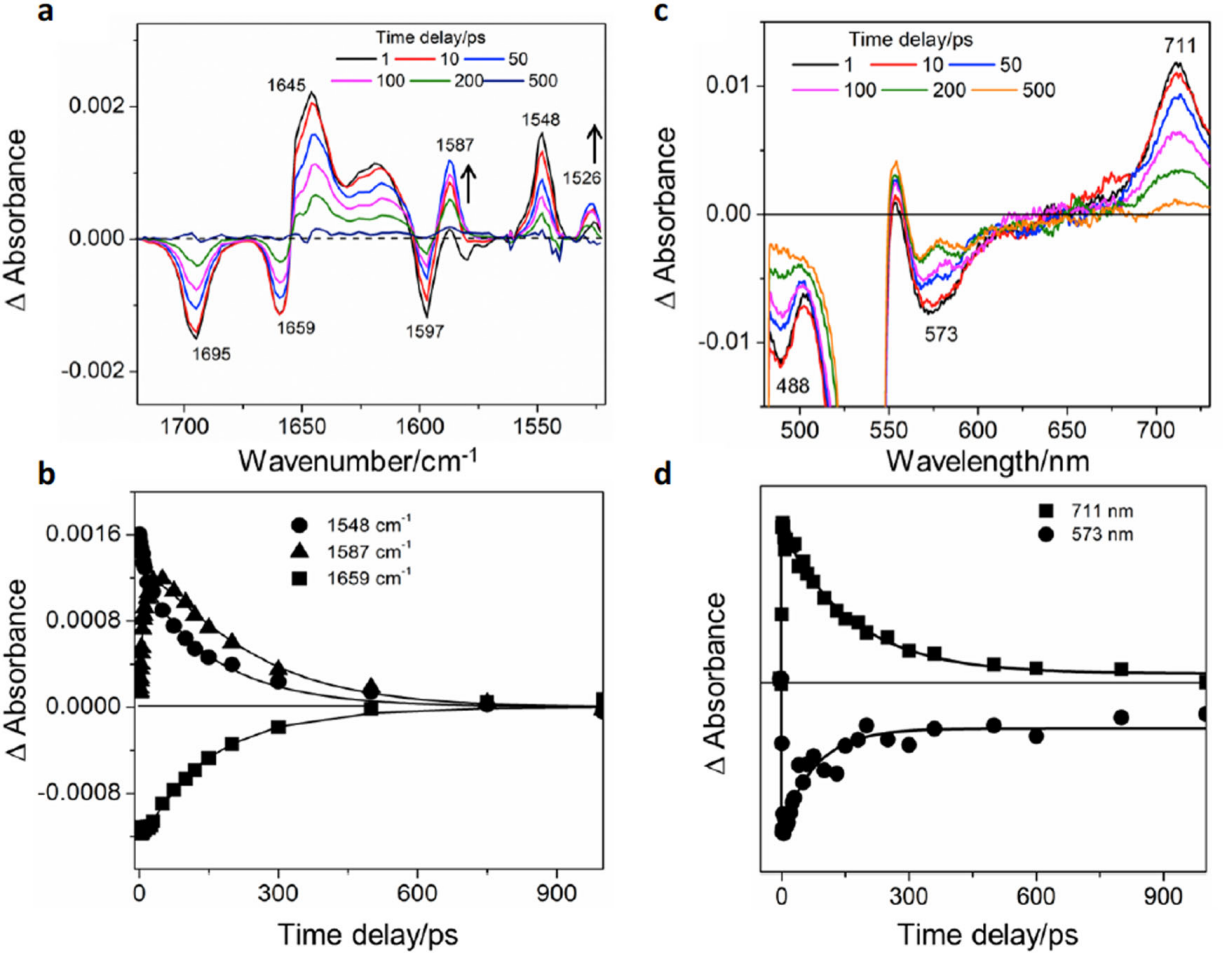
Time resolved spectroscopic studies of [PDI-(P5A)_2_]^2+^. The TRIR and TA results of [PDI-(P5A)_2_]^2+^ probed after 532 nm photoexcitation in CH_2_Cl_2_, showing (**a**) the ps-TRIR spectra at selected time delays. The arrows indicate the formation of PDI^•−^ radical anion bands. **b** Amplitude kinetics for selected peak frequencies with fit; the triangles, solid squares, and circles show experimental points; the continuous lines show the best-fit lines. **c** ps-TA spectra at selected time delays. The 573 nm band is due to stimulated emission and the 488 nm band is due to ground state bleach. **d** Kinetic traces with fit for selected probe wavelengths. The squares, and circles show experimental points. The continuous lines show the best-fit lines.

The TRIR spectrum obtained 1 ps following excitation (532 nm) of [3]rotaxane [PDI-(P5A)_2_]^2+^ shows the formation of an excited, PDI-based, singlet state, ^1^PDI, with peaks at 1645, 1617, and 1548 cm^−1^ (Fig. 5a). The spectrum can be assigned by comparison with previous studies^38^. After 10 ps, new bands grow, at the same rate as ^1^PDI partially decays (*τ*_1_ = 9 (±2) ps), at 1587 and 1526 cm^−^^1^ accompanied by new features slightly red-shifted at 1645 and 1617 cm^−^^1^. These band positions are consistent with the first reduced species observed in the IR spectroelectrochemistry (Fig. 4a) and are assigned to the formation of a PDI^•−^ species. To examine the picosecond dynamics more qualitatively, TRIR data was analysed by global fitting using a multiexponential function. The observed spectral changes and kinetics are well reproduced with three components having time constants (*τ*_1_ = 9 ps, *τ*_2_ = 100 ps, and *τ*_3_ = 180 ps, Fig. 5b).

**Fig. 6:**
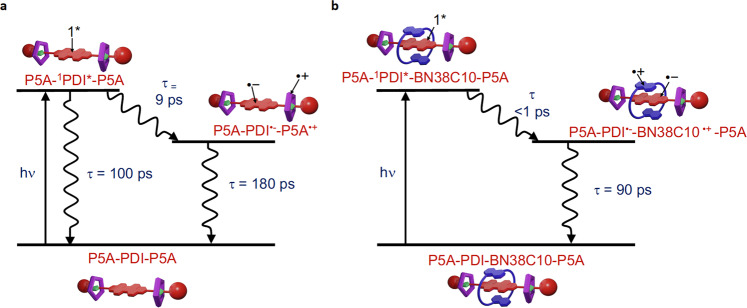
Proposed decay pathways for [PDI-(P5A)_2_]^2+^ and [PDI-BN38C10-(P5A)_2_]^2+^. **a** Following excitation [PDI-(P5A)_2_]^2+^ forms a ^1^PDI excited state which undergoes partial charge separation in 9 ps to form the PDI^•−^ species. The ^1^PDI decays via internal conversion (IC) after 100 ps, with the PDI^•−^ species undergoing recombination back to the ground state after 180 ps. **b** When [PDI-BN38C10-(P5A)_2_]^2+^ is photoexcited it quickly undergoes charge separation from a ^1^PDI in less than 1 ps, forming the PDI^•−^ species which undergoes recombination back to the ground state after 90 ps.

Immediately after photoexcitation, the decay with *τ*_1_ = 9 ps is associated with partial decay of the ^1^PDI and formation of PDI^•−^ and is attributed to the charge transfer rate from P5A to ^1^PDI. The intermediate time constant, *τ*_2_ = 100 ps, is associated with partial bleach recovery concurrent with ^1^PDI decay to the parent. The longest time constant *τ*_3_ = 180 ps is assignable to the lifetime of PDI^•−^. The TA results further support the TRIR observations. A broad band centred at 711 nm (Fig. 5c), which narrows after 9 ps, can be assigned to the PDI^•−^ species formation, an observation again supported by the spectroelectrochemistry (Fig. 3 and Supplementary Fig. 5). A stimulated emission band is also seen at 573 nm which decays at the same rate (*τ* = 95 ps) as the slower decay of ^1^PDI observed in the TRIR experiments. Combining TRIR and TA results, a proposed decay pathway of [PDI-(P5A)_2_]^2+^ is shown in Fig. 6. Following excitation, the ^1^PDI excited state undergoes partial charge separation to form a PDI^•−^ species in 9 ps, following the donation of an electron from either one of the P5A rings. Whilst the ^1^PDI undergoes internal conversion (IC) back down to the ground state in 100 ps, the PDI^•−^ species undergoes recombination (RC) to the ground state in 180 ps. The coexistence of a ^1^PDI and charge-separated state has been observed previously by Wasielewski and co-workers^39^.

**Fig. 7:**
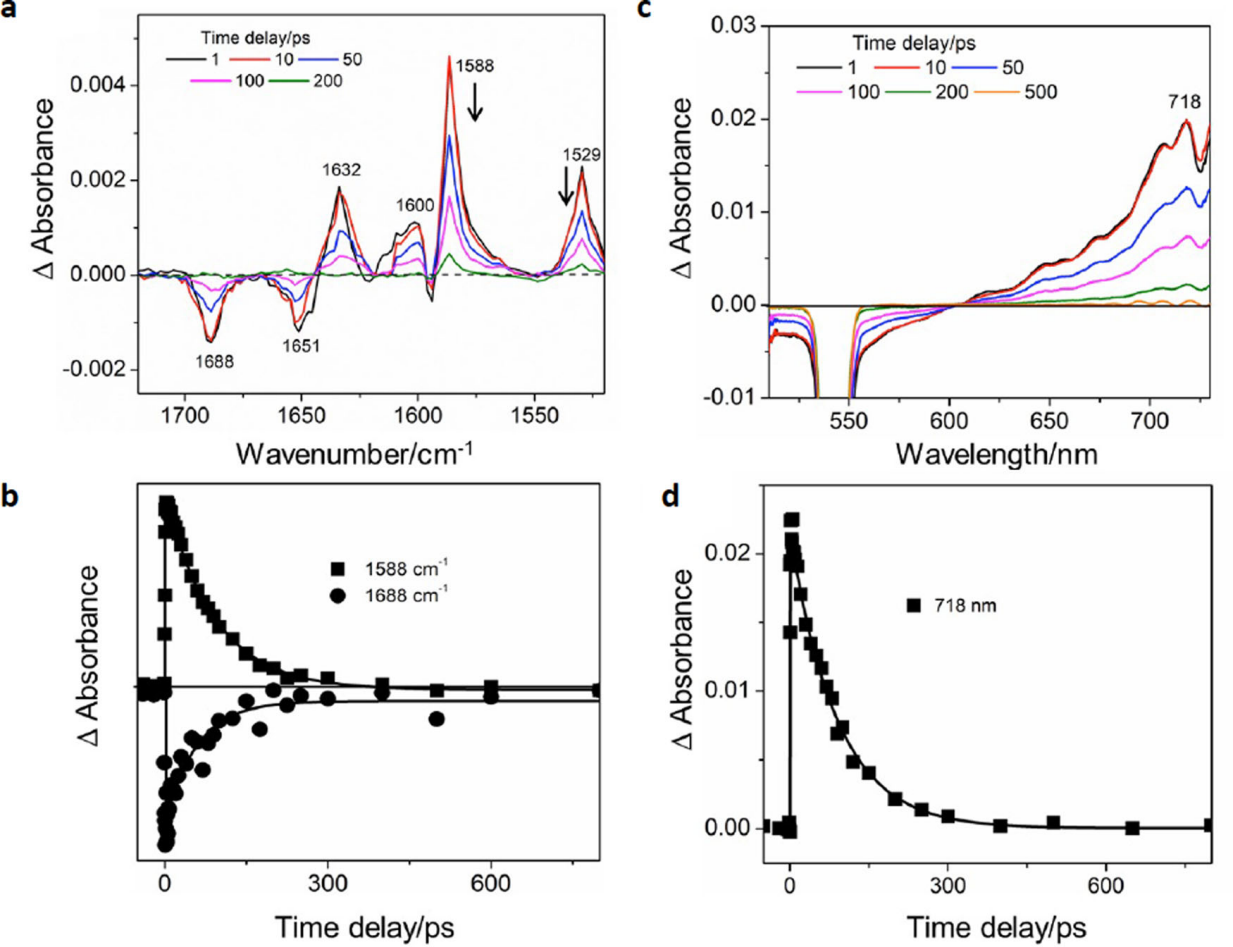
Time resolved spectroscopic studies of [PDI-BN38C10-(P5A)_2_]^2+^. TRIR and TA results of [PDI-BN38C10-(P5A)_2_]^2+^ probed after 532 nm photoexcitation in CH_2_Cl_2_, showing (**a**) the ps-TRIR spectra at selected time delays. The arrows indicate decay of PDI^•−^ bands which are formed within <1 ps. **b** Amplitude kinetics for selected peak frequencies with fit. The squares and circles show experimental points. The continuous lines show the best-fit lines. **c** ps-TA spectra at selected time delays. **d** Kinetic trace with fit for selected probe wavelength. The squares show experimental points. The continuous line shows the best-fit line.

Similar TRIR and TA experiments were performed on the [4]rotaxane, [PDI-BN38C10-(P5A)_2_]^2+^. In this case only formation of the PDI^•−^ species is observed after initial excitation (1632, 1600, 1588, and 1529 cm^−^^1^), (Fig. 7a). The absence of ^1^PDI features suggests that charge separation occurs rapidly (<1 ps). The PDI^•−^ species is seen to decay, with a lifetime of 90 ps, accompanied by the parent recovery (Fig. 7b). PDI^•−^ formation is also observed in the TA spectra, with a transient at 718 nm (Fig. 7c), which decays with a lifetime of 90 ps (Fig. 7d) and no stimulated emission was observed in the TA for [PDI-BN38C10-(P5A)_2_]^2+^. The proposed decay pathway for [PDI-BN38C10-(P5A)_2_]^2+^ is shown in Fig. 6. Thus, following photoexcitation, a very fast charge separation occurs (<1 ps), where full population of the charge-separated state is observed, forming a PDI^•−^ species, which undergoes recombination back to the ground state after ca. 90 ps. Due to the fast kinetics observed for [PDI-BN38C10-(P5A)_2_]^2+^ and the close proximity of the BN38C10 macrocycle to the PDI core, it is likely that the electron donation occurs from the BN38C10 macrocycle.

Synthesis of homo[3]rotaxane and hetero[4]rotaxanes from redox and photoactive components allows control over energy transfer pathways in molecular assemblies. Our strategy involves the use of relatively simple components including macrocycles P5A and BN38C10 that are widely used in supramolecular chemistry. Using a rod, or thread, that allows organisation of these macrocycles on specific recognition sites, imidazolium or PDI respectively, enables the targeted organisation of donor and acceptor groups in specific sequences. A combination of (spectro)electrochemical and time-resolved spectroscopic studies demonstrate that whereas the homo[3]rotaxane, [PDI-(P5A)_2_]^2+^, exhibits the formation of a charge-transfer state in which the P5A molecule acts as a donor and the PDI as acceptor, the hetero[4]rotaxane, [PDI-BN38C10-(P5A)_2_]^2+^, offers an alternative pathway for charge separation, and hence energy transfer. In this latter system, the close proximity of the BN38C10 macrocycle to the PDI enables charge transfer between these two components rather than involving the P5A, despite the P5A possessing a lower energy HOMO. This not only introduces alternative energy transfer pathways but also affects the symmetry in the interlocked molecule. As charge transfer in [3]rotaxane, [PDI-(P5A)_2_]^2+^, occurs between a solitary P5A and PDI desymmetrisation of the rotaxane takes place, with only one P5A being oxidised on the timescale of the charge separation process. In contrast for [4]rotaxane, [PDI-BN38C10-(P5A)_2_]^2+^, as the charge transfer only involves the BN38C10 macrocycle and PDI, the P5A macrocycles remain unperturbed. Thus, our design allows both control over intramolecular energy transfer pathways but also molecular symmetry.

## Methods

All the reagents used were purchased from commercial suppliers and used without further purification. ^1^H, ^13^C, ^19^F, and ^31^P NMR spectra were recorded using a Bruker AV(III)400HD spectrometer. MALDI-TOF MS spectra were recorded on a Bruker Ultraflex III spectrometer using trans-2-[3-(4-tertbutylphenyl)-2-methyl-2-propenylidene]-malononitrile as the matrix. EI M/S spectra were taken using a Bruker Apex IV 4.7 T mass spectrometer and ESI M/S spectra were recorded with a Bruker MicroTOF. Elemental analysis was performed using an automated CE-440 Elemental Analyser. Pillar[5]arene^40^, 2,4,6-trimethylbenzyl iodide^28^, N,N′-bis(8-imidazolyloctyl)-perylene-3,4,9,10-tetracarboxylic diimide^29^, bis(1,5-naphtho)-38-crown-10^35^ and [^*n*^Bu_4_N][BF_4_]^41^ were synthesised according to literature procedures. Column chromatography was performed on Merck silica gel 60 (0.2–0.5 mm, 50–130 mesh).

### Crystallographic studies

#### Single crystal X-ray diffraction studies

Single crystals of [PDI-BN38C10-(P5A)_2_](PF_6_)_2_ were grown from a solution of the compound dissolved in CHCl_3_ containing 1 mM [^*n*^Bu_4_N][BF_4_]. A suitable single crystal was selected and mounted using fomblin film on a micromount. Data were collected on a Fluid Film Devices 3-circle diffractometer equipped with a Dectris Pilatus 2M detector. The crystals were kept at 100(2) K during data collection. Using Olex2^42^, the structures were solved with the ShelXT^43^ structure solution programme using Intrinsic Phasing and refined with the ShelXL^44^ refinement package using Least Squares minimisation.

Crystal Data for C_188_Cl_18_F_12_H_192_N_6_O_24_P_2_ (*M* = 3847.50 g/mol): triclinic, space group *P*-1 (no. 2), *a* = 12.1922(7) Å, *b* = 13.9536(11) Å, *c* = 27.923(3) Å, *α* = 102.330(7)°, *β* = 95.182(6)°, *γ* = 102.574(6)°, *V* = 4482.7(6) Å3, *Z* = 1, *T* = 100(2) K, *μ*(Synchrotron) = 0.347 mm^−1^, *D*_calc_ = 1.425 g/cm3, 19267 reflections measured (2.986° ≤ 2Θ ≤ 31.988°), 4766 unique (*R*_int_ = 0.0984, *R*_sigma_ = 0.0831) which were used in all calculations. The final *R*_1_ was 0.3410 (*I* > 2σ(*I*)) and *wR*_2_ was 0.6948 (all data). Details of dealing with disorder and other refinement are described in the corresponding, deposited cif, CCDC 2114986, but are also described in the Supplementary Information file for convenience.

### Electron diffraction crystal structure determination

Electron diffraction measurements were collected using a Rigaku Synergy-ED equipped with a Rigaku HyPix-ED detector optimised for operation in the Micro-ED experimental setup^45^. The sample consisted of flake-like crystallites with approximately 100 nm thickness. A total of ten data sets were collected. The single data sets were measured at room temperature (293(2) K) from different crystallites with a wavelength of 0.0251 Å. The single measurements took approximately 10–15 min each, resulting in a total experiment time of 2 h. Due to the low symmetry space group, the data of the single measurements were weak and incomplete with completeness varying between 50 and 70%, depending on the scan lengths. Nevertheless, structure solutions with the single measurements were possible. For improved data quality, a total of 9 measurements were merged, resulting in a comprehensive data set with a resolution limit of 1.0 Å. The improvement of data quality indicators is shown in Table S3 in Supplementary Information, accompanied by further details and examples of electron diffraction patterns.

The structure was solved with intrinsic phasing (SHELXT42), revealing the molecular structure, and refined using SHELXL^44^ in Olex2^42^ in a mostly unconstrained fashion. Details of dealing with disorder and other refinement are described in the corresponding, deposited cif, CCDC 2114987. The PF_6_^−^ counter ion was placed on a maximum in the residual density map. However, due to severe disorder no single atom positions were observable and the molecule was constrained as a rigid group. The presence of PF_6_^−^ was confirmed by NMR results. Basic structural features could be determined from the structure solutions of single data sets. By merging multiple data sets, more complete, significant, and redundant data were created and resulted in a high-quality structure refinement.

Crystal Data for C_192_H_210_F_12_N_6_O_34_P_2_ (*M* = 3435.72 g/mol): triclinic, space group *P*-1 (no. 2), *a* = 12.761(17) Å, *b* = 14.39(2) Å, *c* = 29.10(5) Å, *α* = 99.59(15)°, *β* = 96.75(14)°, *γ* = 103.42(12)°, *V* = 5056(14) Å^3^, *Z* = 1, *T* = 293(2) K, *D*_calc_ = 1.128 g/cm^3^, 97091 reflections measured (0.202° ≤ 2Θ ≤ 1.438°), 9201 unique (*R*_int_ = 0.3529, *R*_sigma_ = 0.1495) which were used in all calculations. The final *R*_1_ was 0.2607 (*I* > 2σ(*I*)) and *wR*_2_ was 0.6034 (all data).

### Electrochemical methods

#### Cyclic voltammetry

Cyclic voltammetry was carried out using an Autolab PGSTAT20 potentiostat under an argon atmosphere using a three-electrode arrangement in a single compartment cell. Glassy carbon was used as the working electrode, platinum wire as the secondary electrode, and a saturated calomel reference electrode, chemically isolated from the test solution via a fritted bridge tube containing electrolyte solution, in the cell. An analyte concentration of 1 mM was used with [^*n*^Bu_4_N][BF_4_] (400 mM) as a supporting electrolyte. Redox potentials are referenced to the ferrocenium/ferrocene couple, which was implemented as an internal reference. No compensation was applied for internal resistance.

#### Spectroelectrochemistry

UV/vis spectroelectrochemical measurements were performed using an optically transparent quartz electrochemical cell, with a 0.5 mm path length. A three-electrode configuration of a platinum/rhodium gauze working electrode, platinum wire secondary electrode, and a saturated calomel reference electrode (chemically isolated via a fritted bridge tube) were used in the cell. The potential at the working electrode was regulated with a Sycopel Scientific Ltd DD10M potentiostat and the spectra recorded with a Perkin Elmer 16 spectrophotometer. Temperature control was achieved with a stream of chilled nitrogen gas (cooled by passing through a tube submerged in liquid nitrogen) across the surface of the cell, adjusting the flow rate as necessary in response to a temperature sensor (±0.1 °C). [^*n*^Bu_4_N][BF_4_] (400 mM) was used as the supporting electrolyte for the experiments.

#### Bulk electrolysis and electron paramagnetic resonance spectroscopy

Bulk electrolysis was performed under an argon atmosphere at 0 °C in a two-component cell: a platinum/rhodium gauze working electrode and secondary electrode are separated by a glass frit. A saturated calomel reference electrode was bridged to the test solution through a vycor frit, oriented at the centre of the working electrode. The working electrode compartment, containing analyte (1 mM), was stirred rapidly with a magnetic stirrer bar during electrolysis. [^*n*^Bu_4_N][BF_4_] (400 mM) was used as the supporting electrolyte for the experiments. After electrolysis was completed, the prepared solution was transferred by cannula to a quartz EPR tube for analysis on a Bruker EMX spectrometer. Solution phase (fluid) spectra were recorded at room temperature. Spectra were simulated using WIN EPR SimFonia software.

### Time-resolved spectroscopy

TRIR spectroscopy was carried out at the University of Nottingham and the apparatus is based on the PIRATE facility at the Rutherford Appleton Laboratory, which has been described previously^46^. Briefly, a commercial Ti:sapphire oscillator (MaiTai)/regenerative amplifier system (Spitfire Pro, Spectra Physics) provides 800 nm, 100 fs, 1 kHz, 2 mJ pulses. Half of the output is used to pump a TOPAS-C (Light Conversion) to produce pump pulses at 532 nm. The other part of the amplifier output is used to pump another TOPAS to generate tunable mid-IR pulses. A portion of the IR pulse is reflected onto a single-element mercury cadmium telluride (MCT) detector (Kolmar Technology) to serve as a reference, while the remainder serves as the probe beam, which is focused and overlaps with the pump beam at the sample position. The 532 nm pump is optically delayed (up to 2 ns) by a translation stage (LMA Actuator, Aerotech) and focused onto the sample with a quartz lens. The polarisation of the pump pulse is set at the magic angle (54.7°) relative to the probe pulse to avoid rotational diffusion. The focus spot of the probe beam is adjusted to be slightly smaller than that of the pump beam to ensure full overlap with the pump beam. A rotating chopper wheel (Thorlabs) blocked every other pump pulse, producing difference spectra with and without photoexcitation. The broad-band-transmitted probe pulse is dispersed with a 250 mm IR spectrograph (DK240, Spectra Product) with a 150 grooves/mm grating, and detected with an MCT array detector (Infrared Associates) consisting of 128 elements. Signals from the array detector elements and the single-element detector are amplified with a 144-channel amplifier and digitised by a 16-bit analogue-to-digital converter (IR-0144, Infrared Systems Development Corp.).

TA measurements are based on a similar pump-probe method as the TRIR. The pump beam is obtained in the same way as above. The probe beam is a pulsed white light continuum, generated by focusing a small amount of the 800 nm output onto 3 mm thick CaF_2_ disk. The white light beam is split into two parts. One part passes through the sample and spatially overlapped with the pump beam. Another part serves as a reference to the probe beam fluctuations. The polarisation of the pump pulse is set at the magic angle (54.7°) relative to the probe pulses. The sample and probe were focused into a spectrograph (Acton, USA) and detected with a linear 1024 CCD array detector (Entwicklungsbüro Stresing). The pump beam size (≥400 μm diameter) is larger than the probe spot (≥200 μm diameter). A Harrick flowing solution cell with 2 mm thick CaF_2_ windows is mounted on a motorised cell mount, which moves the cell in *x* and *y* dimensions rapidly and continuously.

## Supplementary information

Supplementary Information

Peer Review File

## Data Availability

The authors declare that all characterisation data generated in this study are provided in the Supplementary Information or within the main manuscript. The X-ray crystallographic coordinates for structures reported in this study have been deposited at the Cambridge Crystallographic Data Centre (CCDC), under deposition numbers 2114986 (X-ray) and 2114987 (electron diffraction). These data can be obtained free of charge from The Cambridge Crystallographic Data Centre via www.ccdc.cam.ac.uk/data_request/cif.
