# Supplementary material for: Selective photoinduced charge separation in perylenediimide-pillar[5]arene rotaxanes
Source: Nat Commun. 2022 Jan 20;13:415. doi: 10.1038/s41467-022-28022-3 (PMC8776946; doi:10.1038/s41467-022-28022-3)
Supplement: Supplementary file 1 — Supplementary Information [file 41467_2022_28022_MOESM1_ESM.pdf]

Selective Photoinduced Charge Separation in Perylenediimide-Pillar[5]arene Rotaxanes

Nicholas Pearce, Katherine E. A. Reynolds, Surajit Kayal, Xue Z. Sun, E. Stephen Davies, Ferdinando Malagreca, Christian J. Schürmann, Sho Ito, Akihito Yamano, Stephen P. Argent, Michael W. George and Neil R. Champness

Supplementary Information Table of Contents

Experimental Details including Synthesis	Page 1
Details of Crystallographic Experiments, X-ray and Electron Diffraction structures	Page 5
Additional UV-vis Spectra and Spectroelectrochemistry and Data	Page 8
Additional EPR Spectra and Data	Page 13
References	Page 14

Further Experimental Details

Synthesis

All the reagents used were purchased from commercial suppliers and used without further purification. ^{1}H, ^{13}C, ^{19}F and ^{31}P NMR spectra were recorded using a Bruker AV(III)400HD spectrometer. MALDI-TOF MS spectra were recorded on a Bruker Ultraflex III spectrometer using trans-2-[3-(4-tertbutylphenyl)-2-methyl-2-propenylidene]-malononitrile as the matrix. EI M/S spectra were taken using a Bruker Apex IV 4.7 T mass spectrometer and ESI M/S spectra were recorded with a Bruker MicroTOF. Elemental analysis was performed using an automated CE-440 Elemental Analyser. 2,4,6-trimethylbenzyl iodide,[1] N,N'-bis(8-imidazolyloctyl)-perylene-3,4,9,10-tetracarboxylic diimide,[2] bis(1,5-naphtho)-38-crown-10[3] and [^{n}Bu$_4$N][BF$_4$][4] were synthesised according to literature procedures, following all procedures precisely. Column chromatography was performed on Merck silica gel 60 (0.2-0.5 mm, 50 - 130 mesh).

Pillar[5]arene (P5A) : A flame dried and N_2 purged flask was charged with 1,4-dimethoxybenzene (10.0 g, 72 mmol) and paraformaldehyde (6.50 g, 216 mmol) and dry CH_2Cl_2 (750 mL) was added via cannula. The mixture was stirred vigorously for 10 min before adding anhydrous iron(III) chloride (1.99 g, 12.2 mmol) was added to form a dark green solution. The solution was stirred under N_2 for 3 hours, after which time the mixture was extracted in portions with water (3 x 600 mL). The combined organic extracts were concentrated under reduced pressure and silica (20 g) was added and the remaining CH_2Cl_2 removed under reduced pressure. The adsorbed mixture was purified via column chromatography (silica, CH_2Cl_2) to afford 1,4-dimethoxypillar[5]arene (2.41 g, 16%) as a white powder. The characterisation data was identical to that provided by Ref 5.

[PDI-(P5A)$_2$](I)$_2$: *N*,*N*'-bis(8-imidazolyloctyl)-perylene-3,4,9,10-tetracarboxylic diimide (47 mg, 63 µmol) and pillar[5]arene (188 mg, 250 µmol) were dissolved in a minimum quantity of $CHCl_3$ (1 mL), sonicated for 10 min and cooled to -10 °C in an ice/salt bath. 2,4,6-Trimethylbenzyl iodide (109 mg, 360 µmol) was added to the solution and the mixture was stirred for 16 h under the exclusion of light. The reaction mixture was purified by column chromatography (silica, 94:6 $CHCl_3$:MeOH) to give the product as a red powder (140 mg, 80%). ^{1}H NMR (400 MHz, $CDCl_3$) δ ppm 8.70 - 8.83 (m, 8 H), 8.27 (s, 2 H), 7.91 (s, 2 H), 7.01 (s, 4 H), 6.91 (s, 10 H), 6.75 (s, 10 H), 6.15 (s, 2 H), 6.00 (d, *J*=15.2 Hz, 2 H), 5.80 (d, *J*=15.3 Hz, 2 H), 4.19 - 4.31 (m, 4 H), 3.80 (s, 30 H), 3.75 (s, 6 H), 3.78 (s, 4 H), 3.67 (s, 4 H), 3.70 (s, 6 H), 3.62 (s, 30 H), 2.45 (s, 12 H), 2.37 (s, 6 H), 1.74 - 1.82 (m, 4 H), 1.27 - 1.35 (m, 4 H), 0.91 - 1.01 (m, 4 H), 0.77 (dt, *J*=15.9, 8.0 Hz, 4 H), -0.26 - -0.09 (m, 4 H), -1.26 - -1.09 (m, 4 H), -1.65 - -1.40 (m, 4 H).

^{13}C NMR (126 MHz, $CDCl_3$) δ ppm 163.34, 151.05, 150.34, 139.59, 138.10, 134.82, 132.60, 131.52, 129.87, 129.68, 129.38, 128.79, 126.60, 126.49, 123.44, 123.25, 123.00, 120.56, 115.73, 113.60, 57.92, 55.64, 48.37, 47.67, 40.66, 30.55, 29.06, 28.78, 28.28, 27.76, 25.78, 25.43, 21.14, 19.97. MS (MALDI)$^+$ m/z 2640.

[PDI-(P5A)$_2$](PF$_6$)$_2$: [PDI-(P5A)$_2$](I)$_2$ (140 mg, 53 µmol) was dissolved in ethanol (20 mL) and heated to 60 °C. NH_4PF_6 (385 mg, 2.4 mmol) was added and the solution stirred for 30 min before cooling to room temperature. The solution was transferred to centrifuge tubes and the precipitate collected by centrifugation, washing two times with methanol to yield the product as a red powder (87 mg, 60%). ^1H NMR (400 MHz, $CDCl_3$) δ ppm 8.60 - 8.77 (m, 8 H), 7.36 (s, 2 H), 7.28 (s, 2 H), 7.05 (s, 4 H), 6.92 (s, 10 H), 6.72 (s, 10 H), 5.91 (t, *J*=1.6 Hz, 2 H), 5.43 - 5.59 (m, 4 H), 4.27 (t, *J*=7.5 Hz, 4 H), 3.80 (s, 30 H), 3.78 (s, 4 H), 3.75 (s, 6 H), 3.69 (s, 6 H), 3.65 (s, 4 H), 3.60 (s, 30 H), 2.42 (s, 12 H), 2.38 (s, 6 H), 1.82 (dt, *J*=15.1, 7.3 Hz, 4 H), 1.41 (dt, *J*=15.3, 7.7 Hz, 4 H), 1.00 (dt, *J*=15.7, 7.7 Hz, 4 H), 0.44 - 0.62 (m, 4 H), 0.12 - 0.28 (m, 4 H), -0.96 - -0.78 (m, 4 H), -1.81 - -1.56 (m, 4 H). ^{13}C NMR (126 MHz, $CDCl_3$) δ ppm 163.18, 151.14, 150.26, 140.05, 137.95, 134.42, 131.97, 131.30, 130.03, 129.86, 128.96, 128.85, 126.08, 125.39, 123.40, 122.89, 121.53, 121.32, 115.96, 113.61, 57.90, 55.65, 47.94, 47.32, 40.45, 30.78, 29.01, 28.70, 28.51, 27.77, 25.95, 25.27, 21.11, 19.49. ^{19}F NMR (375 MHz, $CDCl_3$) δ ppm -72.58 (d, $^1J_{FP}$=711 Hz, 12 F). ^{31}P NMR (162 MHz, $CDCl_3$) δ ppm -144.12 (sept, $^1J_{PF}$=711 Hz, 2 P). HRMS (MALDI)$^+$ m/z 2658.2160 ($C_{156}H_{172}N_6O_{24}PF_6$ [M^{2+} + PF_6^-]$^+$ requires 2658.2059).

[PDI-BN38C10-(P5A)$_2$](PF$_6$)$_2$: *N*,*N'*-bis(8-imidazolyloctyl)-perylene-3,4,9,10-tetracarboxylic diimide (53 mg, 71 µmol) was added to a solution of bis(1,5-naphtho)-38-crown-10 (80 mg, 126 µmol) in $CHCl_3$ (1 mL) forming a purple solution, which was sonicated for 10 min. Pillar[5]arene (160 mg, 213 µmol) was added and the solution further sonicated for 10 min, and cooled to -10 °C in an ice/salt bath. 2,4,6-Trimethylbenzyl iodide (107 mg, 355 µmol) was added to the solution and the mixture was stirred for 16 h under the exclusion of light. The reaction mixture was passed through a silica column, eluting the purple fraction with 94:6 $CHCl_3$:MeOH to remove unreacted reagents, affording the crude rotaxane product as a purple powder. This powder (150 mg) was suspended in ethanol (20 mL) and heated to 70 °C before the addition of NH_4PF_6 (150 mg, 920 µmol). The reaction mixture was stirred for 30 min, cooled and the precipitate collected by centrifugation, washing two times with methanol. The pellet was further purified by preparative TLC (silica, 95:5 $CHCl_3$:MeOH) and column chromatography (alumina, 90:10 $CHCl_3$:MeOH) in an attempt to separate the purple band of the [4]-rotaxane product, [PDI-BN38C10-(P5A)$_2$](PF$_6$)$_2$, from a leading red band, assumed to be [PDI-(P5A)$_2$](PF$_6$)$_2$. The anion exchange reaction was repeated, and the product was purified by layering a 1:1 mixture of hexane and MeOH over a $CHCl_3$ solution of the compound in a centrifuge tube, collecting the purple crystals soon after formation by filtration under reduced pressure, washing with cold hexane. The filtered solvent could be removed and the crystallisation process repeated until the mother liquor was noticeably more red, at which point further crystallisation could not selectively precipitate the desired [PDI-BN38C10-(P5A)$_2$](PF$_6$)$_2$. The yield of the analytically pure [PDI-BN38C10-(P5A)$_2$](PF$_6$)$_2$ was 16 mg (6.5%). ^1H NMR (500 MHz, $CDCl_3$) δ ppm 8.42 - 8.60 (m, 8 H), 7.46 (s, 2 H), 7.29 (s, 2 H), 7.05 (s, 4 H), 6.97 (s, 10 H), 6.70 - 6.78 (m, 14 H), 6.14 (t, *J*=7.8 Hz, 4 H), 5.89 (s, 2 H), 5.69 (dt, *J*=7.4, 3.5 Hz, 4 H), 5.57 (d, *J*=14.9 Hz, 2 H), 5.50 (d, *J*=14.9 Hz, 2 H), 4.28 - 4.39 (m, 4 H), 4.05 - 4.11 (m, 8 H), 4.00 - 4.05 (m, 8 H), 3.90 - 3.94 (m, 8 H), 3.88 (s, 30 H), 3.81 - 3.85 (m, 8 H), 3.77 - 3.81 (m, 10 H), 3.69 (s, 4 H), 3.71 (s, 6 H), 3.61 (s, 30 H), 2.43 (s, 12 H), 2.39 (s, 6 H), 1.96 (dt, *J*=15.2, 7.9 Hz, 4 H), 1.51 - 1.56 (m, 4 H), 1.12 (dt, *J*=15.9, 7.9 Hz, 4 H), 0.46 - 0.56 (m, 4 H), 0.23 - 0.39 (m, 4 H), -0.88 - -0.73 (m, 4 H), -1.79 - -1.55 (m, 4 H). ^{13}C NMR (126 MHz, $CDCl_3$) δ ppm 164.02, 152.83, 151.12, 150.42, 139.94, 138.07, 134.63, 131.82, 130.71, 130.03, 129.87, 128.94, 127.97, 125.66, 125.21, 124.80, 123.81, 122.96, 122.17, 121.83, 121.26, 115.96, 113.70, 113.52, 102.96, 71.47, 71.38, 70.07, 67.61, 57.93, 55.77, 48.16, 47.29, 40.47, 30.98, 29.08, 29.00, 28.73, 28.10, 25.99, 25.15, 21.16, 19.63. ^{19}F NMR (375 MHz, Chloroform-*d*) d ppm -72.60 (d, $^1J_{FP}$=711 Hz, 12 F). ^{31}P NMR (162 MHz, $CDCl_3$) δ ppm -144.16 (sept, $^1J_{PF}$=711 Hz, 2 P). MS HRMS (MALDI)$^+$ m/z 3149.5338 ($C_{192}H_{216}N_6O_{34}$ [M^{2+} + e^-]$^+$ requires 3149.5352).

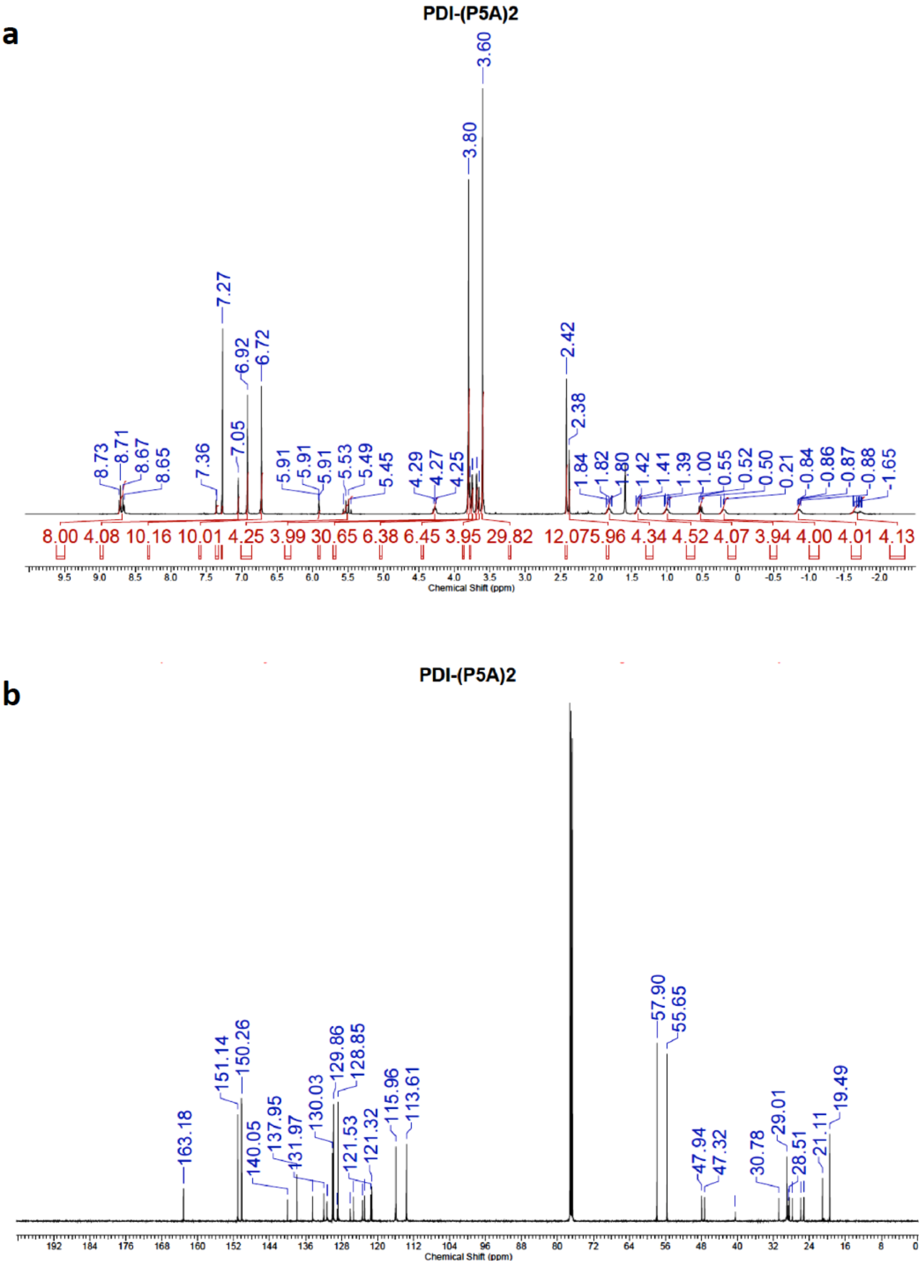

Supplementary Figure 1. a) ^{1}H and b) ^{13}C NMR spectra of $[PDI\text{-}(P5A)_2](PF_6)_2$ recorded in $CDCl_3$.

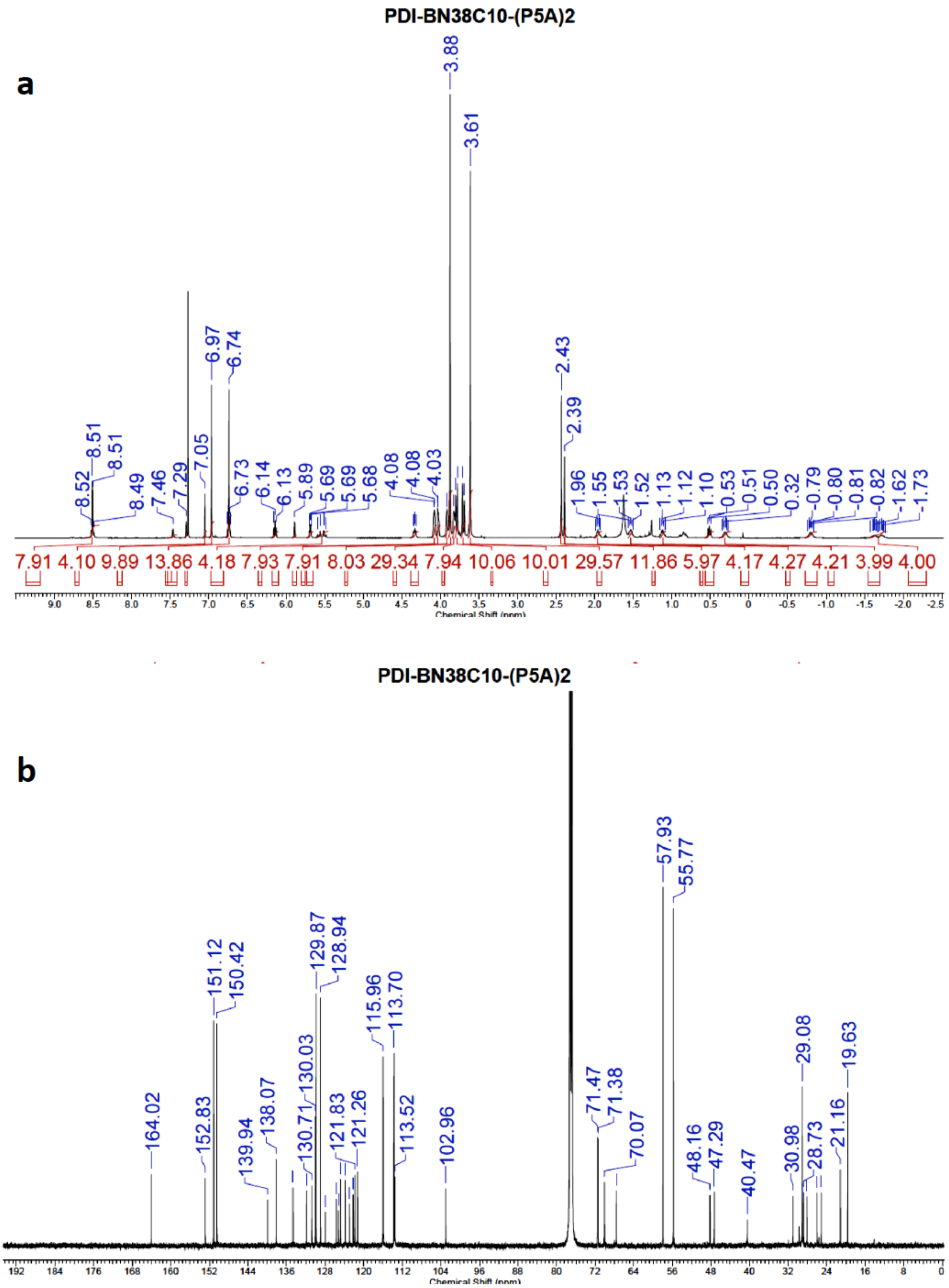

Supplementary Figure 2. a) ^{1}H and b) ^{13}C NMR spectra of [PDI-BN38C10-(P5A)$_2$](PF$_6$)$_2$ recorded in $CDCl_3$.

Crystallographic Details

Details of dealing with disorder in the single crystal structure of [PDI-BN38C10-(P5A)$_2$](PF$_6$)$_2$ determined by X-ray diffraction, and other refinement are described in the corresponding, deposited cif, CCDC 2114986, but are also described herein. The crystals of [PDI-BN38C10-(P5A)$_2$](PF$_6$)$_2$ salt diffracted weakly with a low resolution limit of 1.3 Å despite the use of synchrotron radiation and several attempts at growing better diffracting crystals. The data used in the refinement was truncated to a resolution of 1.25 %A resulting in a modest data to parameter ratio of 13.5. The electron density map supports the chemical identity and gross conformation of the supramolecular assembly but no further detailed geometric information can be drawn from the heavily restrained structure.
The structure was solved with intrinsic phasing (SHELXT) which revealed enough atom positions in all three crystallographically unique residues (axle and two types of macrocycle) to confirm the identity and gross conformation of the assembly. The model was developed by searching for further atoms in the electron density map with some success, however, the electron density in some regions of the structure was too diffuse to locate individual atoms. The diffuse regions corresponded to the more mobile moieties in the structure: ethylene glycol chains, mesityl stopper groups and the methoxy functional groups on the pillararene. In these regions of the structure the expected atoms (based on the synthetic procedure and evidence from NMR and mass spectroscopy) were placed in the expected positions based on the fragments located in the electron density map before being geometrically restrained or constrained to refine to a chemically sensible conformation. The macrocyclic naphthalene moiety, pillararene phenyl moieties and imidazole moiety were constrained to have regular hexagonal and pentagonal ring geometries respectively (AFIX 116, AFIX 66, AFIX 56). The mesityl stopper moiety was refined as a rigid body with coordinated taken from the Olex2 Fragment DB. All other 1,2 and 1,3 atomic distances in the structure were restrained to have target values and conformations using restraints generated by the Grade Web Server which draws on information from the Cambridge Structure Database and quantum mechanical calculations (DFIX, DANG, FLAT) (http://grade.globalphasing.org).
All atoms in the structure were refined with isotropic displacement parameters - the weak low resolution data did not support meaningful refinement of atoms anisotropically. The isotropic displacement parameters of all atoms were refined with a similarity restraint (SIMU). The pillararene methoxymoieties were refined at half chemical occupancy. Peaks in the electron density map indicated it is likely that the pillararene is orientationally disordered with the methoxy moieties located on the other phenyl ring position some of the time, however, the data did not support development of a stable model for this disorder. The methoxy moieties are included in the unit cell contents at full occupancy. An expected PF_6^- counter ion (one per asymmetric unit) was unsurprisingly not found in the electron density map and accounted for, along with three $CHCl_3$ residues, based on the void volume calculated by the solvent mask routine in Olex2. The missing atoms of these residues were included in the unit contents. A solvent mask was calculated and 146 electrons were found in a volume of 623 Å^3 in 2 voids per unit cell. This is consistent with the presence of 2[PF$_6$], 6[CHCl$_3$] per asymmetric Unit which account for 486 electrons per unit cell.

Electron Diffraction Crystal Structure Determination

Electron diffraction measurements were collected using a Rigaku Synergy-ED equipped with a Rigaku HyPix-ED detector optimised for operation in the Micro-ED experimental setup. The sample consisted of flake-like crystallites with approximately 100 nanometer thickness. A total of ten data sets were collected: data sets numbered **880** to **889**. For improved data quality, a total of 9 measurements were merged, resulting in a comprehensive data set with a resolution limit of 1.0 Å. Supplementary Table 3 features the improvement of data quality indicators, i.e. R_{pim}, by the inclusion of additional data. For the ultimate data set, **887** was not included, due to the relatively low intensity.

Supplementary Table 1. Data collection parameter overview.

Data	*Number of frames*	*Scan width (°)*	*Exposure time (s)*	*Total time*
**880**	128	1	5	00:10:51
**881**	126	1	5	00:11:15
**882**	164	0.5	5	00:14:24
**883**	140	1	5	00:12:56
**884**	122	1	5	00:10:21
**885**	122	1	5	00:10:21
**886**	184	0.5	5	00:15:31
**887**	184	0.5	5	00:15:31
**888**	110	1	5	00:09:55
**889**	184	0.5	5	00:15:31

Supplementary Table 2. Data quality statistics overview for all single data collections. All data were processed up to a resolution of 1.0 Å. Point group symmetry: *P*-1. Theoretical #Data: 10581.

**Name**	**Data**	**Comp. %**	**Redund.**	**$<F^2>$**	**$<F^2/\sigma(F^2)>$**	**R_{int}**	**R_{σ}**
**880**	14420	70.5	2.0	163.20	5.51	0.152	0.155
**881**	14319	69.6	2.0	122.55	4.33	0.199	0.199
**882**	8989	44.4	2.0	275.89	9.41	0.099	0.083
**883**	15487	75.2	2.0	219.34	5.38	0.182	0.163
**884**	13688	67.0	2.0	180.33	5.81	0.162	0.160
**885**	13742	66.9	2.0	163.28	5.00	0.130	0.152
**886**	10341	50.6	2.0	130.98	7.03	0.114	0.115
**887**	10101	50.9	2.0	78.64	5.39	0.133	0.131
**888**	12174	59.6	2.0	318.83	7.85	0.138	0.107
**889**	10273	50.5	2.0	286.61	11.94	0.103	0.067

Supplementary Table 3. Data quality statistics overview for merged data. "--" implies, that all intermediate runs were merged. All data were processed up to a resolution of 1.0 Å. Point group symmetry: *P*-1. Theoretical #Data: 10581.

**Name**	**Data**	**# unique**	**Comp. %**	**Redund.**	**$<F^2>$**	**$<F^2/\sigma(F^2)>$**	**R_{int}**	**R_{σ}**	**R_{rim}**	**R_{pim}**
**880**	14420	7313	70.5	2.0	163.20	5.51	0.152	0.155	0.229	0.152
**merg881--880**	27606	7285	68.8	3.8	123.72	5.50	0.225	0.125	0.253	0.137
**merg882--880**	35782	7822	73.9	4.6	100.21	6.29	0.268	0.138	0.295	0.148
**merg883--880**	50036	8570	81.0	5.8	92.42	6.67	0.324	0.167	0.394	0.163
**merg884--880**	62993	8570	81.0	7.4	119.18	7.44	0.292	0.097	0.272	0.117
**merg885--880**	76120	9189	86.8	8.3	106.23	7.62	0.309	0.124	0.318	0.121
**merg886--880**	85793	9200	86.9	9.3	100.47	8.30	0.312	0.121	0.320	0.116
**merg887--880**	93496	9267	87.6	10.1	89.25	7.70	0.328	0.107	0.295	0.119
**merg888--880**	104446	9268	87.6	11.3	93.64	8.44	0.326	0.107	0.296	0.113
**merg889--880**	113620	9268	87.6	12.3	95.97	9.22	0.321	0.099	0.283	0.106
**merg889-888-886--880**	97091	9201	87.0	10.6	106.58	9.00	0.306	0.114	0.309	0.107

Supplementary Table 4. Statistics vs resolution for final data set **merg889-888-886--880** point group symmetry: P-1.

Resolution (Å)	Data	Comp. %	Redund.	<F2>	$<F^2/\sigma(F_2)>$	R_{int}	R_σ	CC 1/2	CC*
28.31- 2.15	8034	87.1	8.7	682.24	49.26	0.099	0.018	0.996	0.999
2.15- 1.71	9658	90	10.1	231.72	20.6	0.207	0.073	0.972	0.993
1.71- 1.50	10036	89.6	10.6	74.74	8.19	0.413	0.221	0.862	0.962
1.50- 1.36	10181	90.1	10.7	40.07	4.34	0.616	0.41	0.575	0.854
1.36- 1.26	10322	90.2	10.8	39.06	4.13	0.622	0.429	0.525	0.830
1.26- 1.19	10153	89.2	10.8	41.29	4.03	0.598	0.419	0.655	0.890
1.19- 1.13	10401	90.1	10.9	28.88	3.00	0.711	0.544	0.316	0.693
1.13- 1.08	10435	90.2	10.9	16.65	1.74	0.829	0.768	0.140	0.496
1.08- 1.04	10271	89.0	10.9	10.39	1.15	0.884	0.883	0.149	0.509
1.04- 1.00	7600	64.1	11.2	8.91	1.09	0.893	0.910	0.055	0.322
**28.31- 1.00**	**97091**	**87.0**	**10.6**	**106.58**	**9.00**	**0.306**	**0.114**	**0.993**	**0.998**

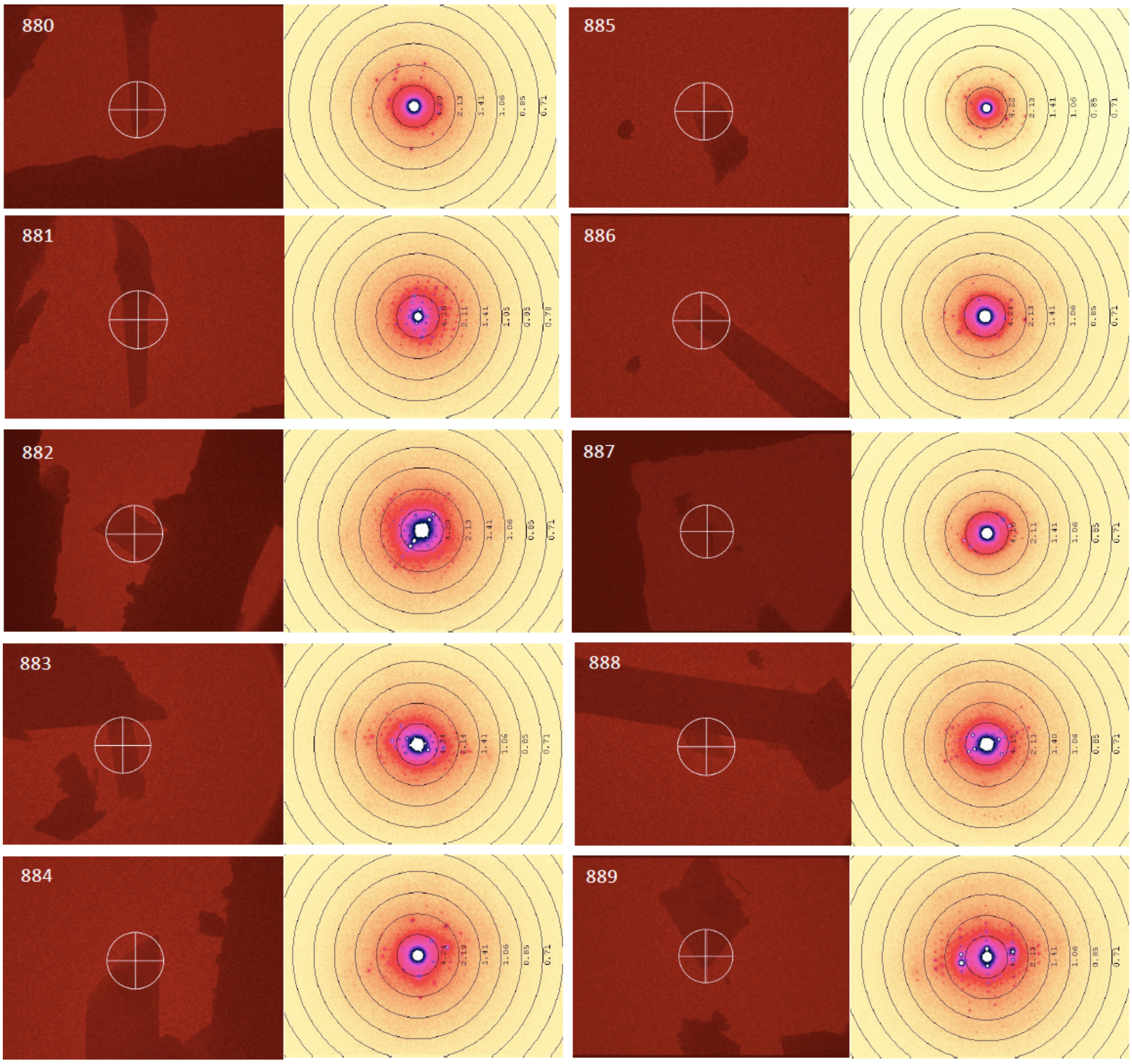

Supplementary Figure 3. Grain snapshots and diffraction images of all data measurements (**880**-**889**). The crosshairs are 4 μm in diameter.

Additional Figures

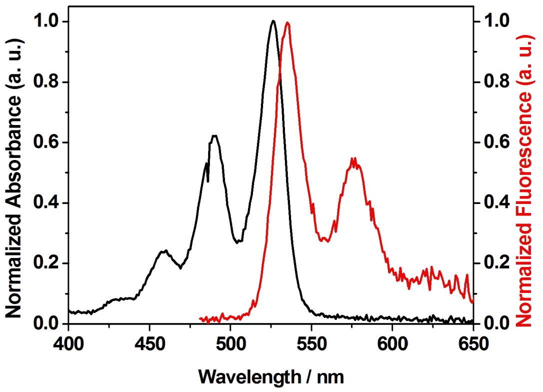

Supplementary Figure 4. Normalized absorbance (black traces) and emission (red traces) spectra of: a) [PDI-(P5A)$_2$](PF$_6$)$_2$ in $CHCl_3$.

Supplementary Table 5 Cyclic Voltammetric data[a]

Compound	1st reduction $E_{1/2}$ / V	2nd reduction $E_{1/2}$ / V	Oxidations / V	ΔEFc$^+$/Fc / V
[PDI-(P5A)$_2$](PF$_6$)$_2$	-0.96 (0.10)	-1.18 (0.07)	+0.75 (0.08)[b]	(0.07)
[PDI-BN38C10-(P5A)$_2$](PF$_6$)$_2$	-1.19 (0.07)	-1.47 (0.07)	+0.77 (0.09)[b]	(0.07)

[a] In CH_2Cl_2 containing [NBu$_4$][BF$_4$] (0.4 M) as supporting electrolyte, at ambient temperature. Potentials quoted at 0.10 Vs^{-1} against $E_{1/2}$ Fc$^+$/Fc used as the internal standard. Values in brackets are ΔE (= $E_p^a - E_p^c$). [b] additional oxidations noted (see Fig. S2)

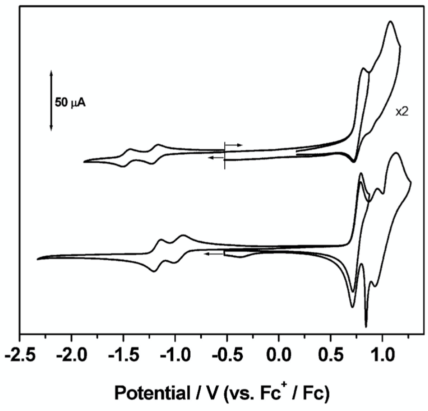

Supplementary Figure 5. Cyclic voltammetry of [PDI-BN38C10-(P5A)$_2$](PF$_6$)$_2$ (upper traces) and [PDI-(P5A)$_2$](PF$_6$)$_2$ (lower traces). In CH_2Cl_2 containing [^{n}Bu$_4$N][BF$_4$] (0.4 M) as supporting electrolyte, at ambient temperature.

Supplementary Table 6. Square Wave Voltammetric data[a]

Compound	1st reduction / V	2nd reduction / V	1st oxidation / V	2nd oxidation / V	3rd oxidation / V
[PDI-(P5A)$_2$](PF$_6$)$_2$	-0.96	-1.17	+0.75	+0.92	+1.02
[PDI-BN38C10-(P5A)$_2$](PF$_6$)$_2$	-1.19	-1.46	+0.76	-[b]	+0.98

[a] In CH_2Cl_2 containing [^{n}Bu$_4$N][BF$_4$] (0.4 M) as supporting electrolyte, at ambient temperature. Potentials reported against $E_{1/2}$ Fc^+/Fc; [b] unresolved shoulder

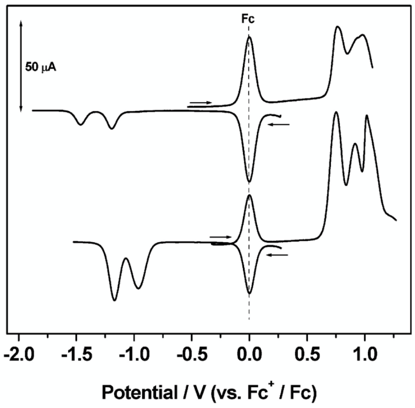

Supplementary Figure 6. Square wave voltammetry of [PDI-BN38C10-(P5A)$_2$](PF$_6$)$_2$ (upper traces) and [PDI-(P5A)$_2$](PF$_6$)$_2$ (lower traces). In CH_2Cl_2 containing [^{n}Bu$_4$N][BF$_4$] (0.4 M) as supporting electrolyte, at ambient temperature.

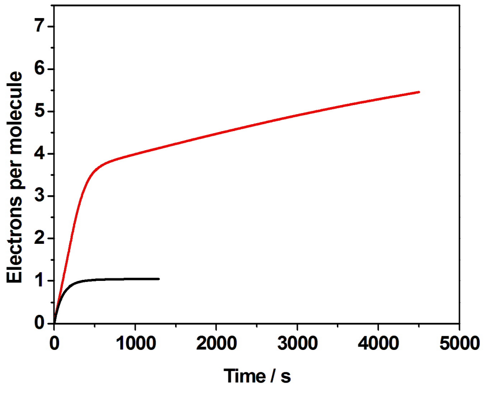

Supplementary Figure 7. Bulk electrolysis profiles for a) [PDI-(P5A)$_2$](PF$_6$)$_2$ showing oxidations (red traces) and first reductions (black traces).

Supplementary Table 7 UV/vis spectroscopic data.[a]

Compound	Neutral λ_{abs}/nm (ε x 10^{-4} /$mol^{-1}dm^3cm^{-1}$)	1st Reduction λ_{abs}/nm (ε x 10^{-4} /$mol^{-1}dm^3cm^{-1}$)	2nd Reduction λ_{abs}/nm (ε x 10^{-4} / $mol^{-1}dm^3cm^{-1}$)	Oxidation λ_{abs}/nm (ε x 10^{-4} / $mol^{-1}dm^3cm^{-1}$)
[PDI-$(P5A)_2$]$(PF_6)_2$	261 (4.2), 294 (5.1), 370 (0.5), 434 (0.7), 459 (2.1), 490 (5.6), 527 (9.4)	262 (4.6), 278 (5.5), 294 (6.9), 352 (0.7), 380 (0.5), 528 (0.4), 680 (4.6), 713 (9.8), 766 (2.3), 797 (5.3), 822 (1.7)[a]	279 (8.1), 292 (10.3), 542 (5.0), 568 (8.4), 589 (6.2), 635 (2.2)	260 (5.4), 300 (5.4), 463 (4.4), 492 (7.7), 530 (11.9)
[PDI-BN38C10-$(P5A)_2$]$(PF_6)_2$	262 (2.7), 294 (4.8), 328 (1.1), 510 (2.5), 545 (2.8)	292 (6.1), 328 (1.2), 725 (7.5), 779 (1.5), 811 (3.8), 863 (0.8)	293 (9.1), 327 (1.4), 572 (6.4), 592 (5.1), 636 (1.5)	297 (4.8), 466 (3.6), 493 (5.3), 530 (7.5)

[a] all processes are reversible under these conditions with the exception of the oxidation of [PDI-BN38C10-$(P5A)_2$]$(PF_6)_2$ (see figure, bands observed at: 260 (4.0), 294 (4.8), 338 (1.4), 460 (1.3), 491 (3.5), 527 (5.7) upon re-oxidation).

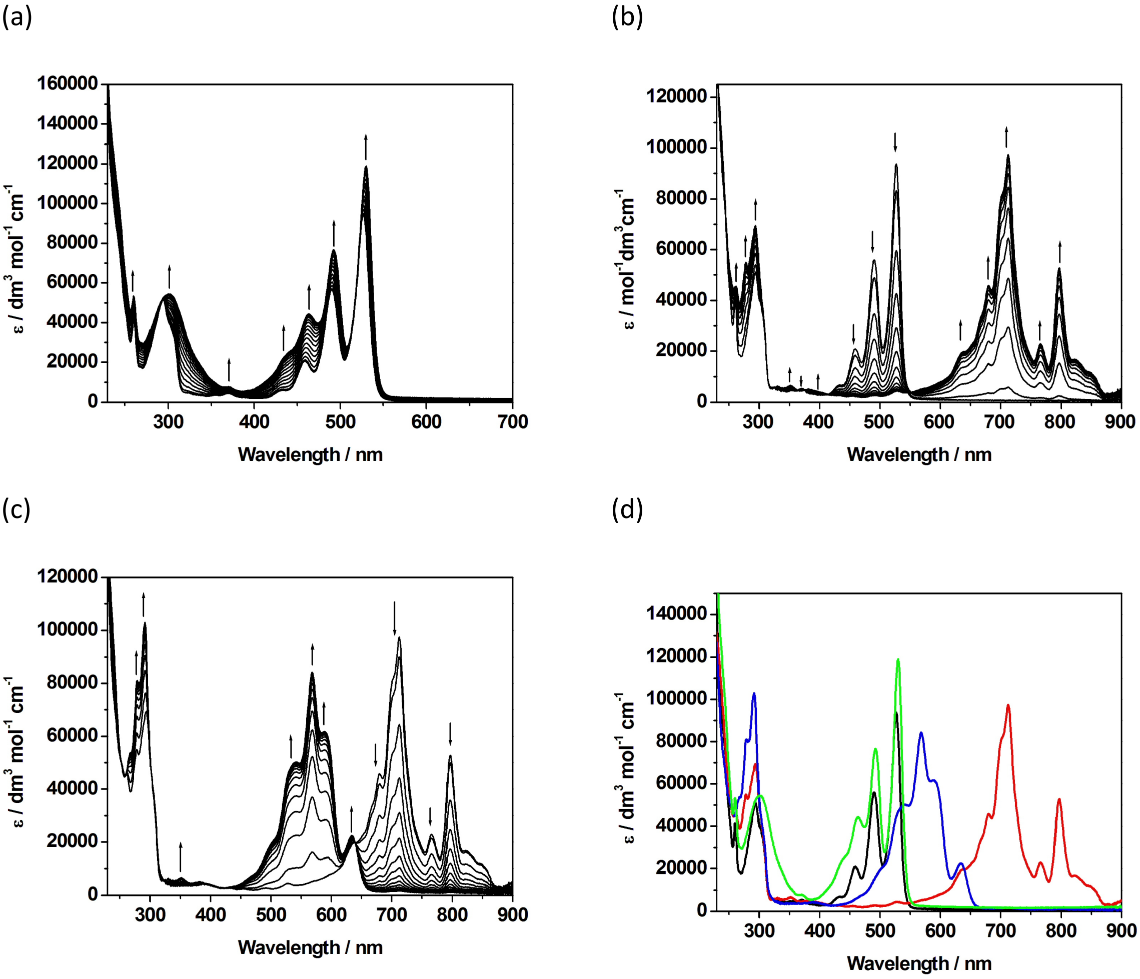

Supplementary Figure 6. UV-vis absorption spectra for $[PDI\text{-}(P5A)_2]^{2+}$ showing: a) the oxidation of $[PDI\text{-}(P5A)_2]^{2+}$ to $[PDI\text{-}(P5A)_2]^{x+}$; b) the inter-conversion of redox states between $[PDI\text{-}(P5A)_2]^{2+}$ and reduction to $[PDI\text{-}(P5A)_2]^{+}$; c) the inter-conversion of redox states between $[PDI\text{-}(P5A)_2]^{+}$ and reduction to $[PDI\text{-}(P5A)_2]$; d) Comparison of spectra of the $[PDI\text{-}(P5A)_2]^{2+}$ in various oxidation states : $[PDI\text{-}(P5A)_2]^{2+}$ - green; $[PDI\text{-}(P5A)_2]^{+}$ - red; $[PDI\text{-}(P5A)_2]$ - blue; $[PDI\text{-}(P5A)_2]^{x+}$ - black. Spectra were recorded in CH_2Cl_2 containing $[^{n}Bu_4N][BF_4]$ (0.4 M) as the supporting electrolyte at 273 K. Arrows indicate the progress of the stated inter-conversion.

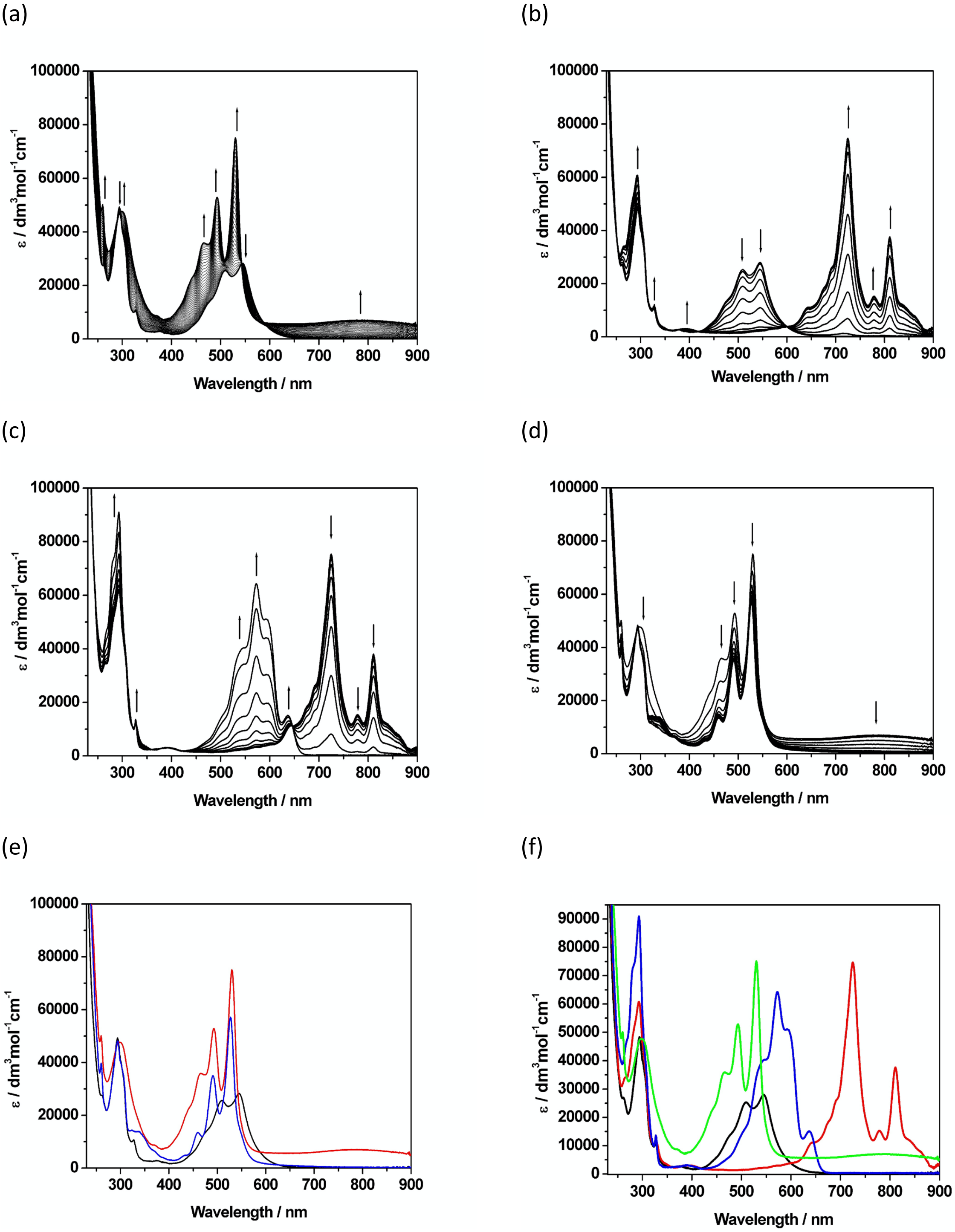

Supplementary Figure 7. UV-vis absorption spectra in [PDI-BN38C10-(P5A)$_2$]$^{2+}$ showing: a) the inter-conversion of redox states between [PDI-BN38C10-(P5A)$_2$]$^{2+}$ and [[PDI-BN38C10-(P5A)$_2$](PF$_6$)$_2$]$^{x+}$; b) the inter-conversion of redox states between [PDI-BN38C10-(P5A)$_2$]$^{2+}$ and monoreduced [PDI-BN38C10-(P5A)$_2$]$^{+}$; c) the inter-conversion of redox states between [PDI-BN38C10-(P5A)$_2$]$^{+}$ and direduced [PDI-BN38C10-(P5A)$_2$]; d) the inter-conversion of redox states between oxidised [PDI-BN38C10-(P5A)$_2$]$^{x+}$ and [PDI-BN38C10-(P5A)$_2$]$^{2+}$; (e) effect of oxidation on [PDI-BN38C10-(P5A)$_2$]$^{2+}$, before oxidation (black line), oxidised (red line), after re-reduction (blue line). (f) d) Comparison of spectra of the [PDI-BN38C10-(P5A)$_2$]$^{2+}$ in various oxidation states : [PDI-BN38C10-(P5A)$_2$]$^{2+}$ - green; [PDI-BN38C10-(P5A)$_2$]$^{+}$ - red; [PDI-BN38C10-(P5A)$_2$] - blue; [PDI-BN38C10-(P5A)$_2$]$^{x+}$ - black. Spectra were recorded in CH_2Cl_2 containing [^{n}Bu$_4$N][BF$_4$] (0.4 M) as the supporting electrolyte at 273 K. Arrows indicate the progress of the stated inter-conversion.

Supplementary Table 8 EPR spectroscopic data[a]

Compound	g_{iso}	a_{iso} / x 10^{-4} cm^{-1}	Linewidth / G	Lineshape
$[PDI-(P5A)_2]^{x+}$ [b]	2.0038	1.700 (2H), 1.14 (4H), 0.650 (8H)*	0.60	Lorentzian
$[PDI-(P5A)_2]^{+}$	2.0033	1.670 (4H), 0.561 (4H), 0.122 (4H), 0.533 (2N)	0.15	Lorentzian
$[PDI-BN38C10-(P5A)_2]^{+}$	2.0033	1.436 (4H), 0.726 (4H), 0.154 (4H), 0.594 (2N)	0.09	Lorentzian

[a] In CH_2Cl_2 containing $[^nBu_4N][BF_4]$ (0.4 M) as supporting electrolyte, at ambient temperature.

[b] Oxidised species

*Position of resolved spectral lines reproduced reasonably by these parameters but significant broadening noted

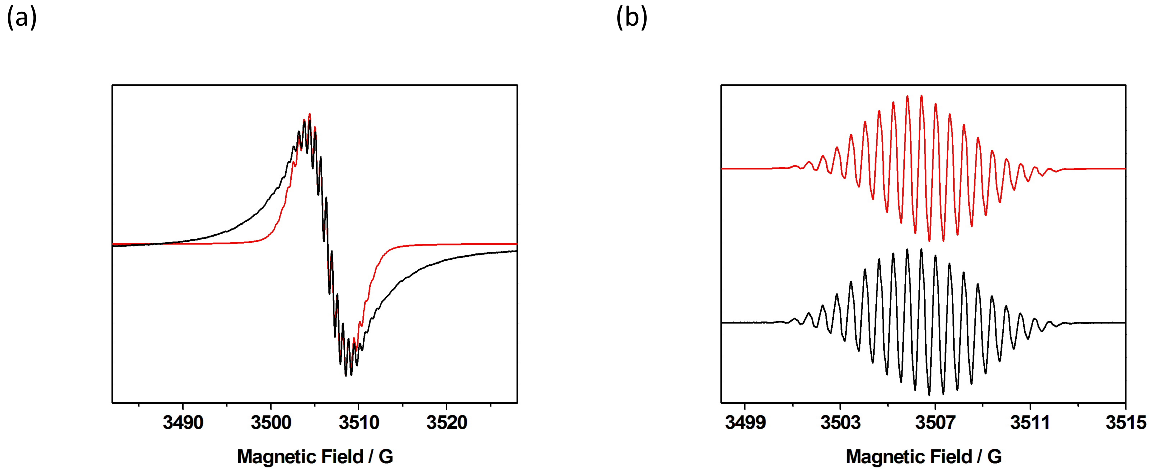

Supplementary Figure 10. Experimental (black trace) and simulated (red trace) EPR spectra of: a) $[PDI-(P5A)_2]^{x+}$, formed by oxidation of $[PDI-(P5A)_2]^{2+}$; b) $[PDI-(P5A)_2]^{+}$, formed by reduction of $[PDI-(P5A)_2]^{2+}$; both as solutions in CH_2Cl_2 containing $[^nBu_4N][BF_4]$ (0.4 M) as supporting electrolyte, at ambient temperature.

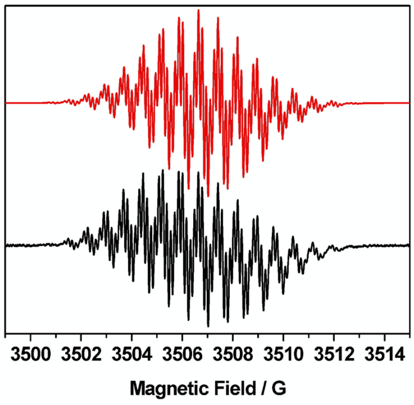

Supplementary Figure 11. Experimental (black trace) and simulated (red trace) EPR spectra of $[PDI-BN38C10-(P5A)_2]^{+}$, formed by reduction of $[PDI-BN38C10-(P5A)_2]^{2+}$, as a solution in CH_2Cl_2 containing $[^nBu_4N][BF_4]$ (0.4 M) as supporting electrolyte, at ambient temperature.

Supplementary References

1. Langer, P. et al. Restricting shuttling in bis(imidazolium)…pillar[5]arene rotaxanes using metal coordination. *Dalton Trans*., **48**, 58-64 (2019).
2. Yang, L. et al. Synthesis and characterisation of rylene diimide dimers using molecular handcuffs. *Chem. Sci*., **10**, 3723-3732 (2019).
3. Hamilton, D. G. et al. Photophysical and Electrochemical Characterisation of the Interactions between Components in Neutral π-Associated [2]Catenanes. *Chem. Eur. J.*, **6**, 608-617 (2000).
4. Kubas, J. Tetrakis(acetonitirile)copper(I) Hexaflurorophosphate. *Inorg. Synth.*, **28**, 68 (1990).
5. Ogoshi, T.; Kanai, S.; Fujinami, S.; Yamagishi, T.; Nakamoto, Y. *para*-Bridged Symmetrical Pillar[5]arenes: Their Lewis Acid Catalyzed Synthesis and Host–Guest Property. *J. Am. Chem. Soc.*, **130**, 5022-5023 (2008).
