# Supplementary material for: Selective photoinduced charge separation in perylenediimide-pillar[5]arene rotaxanes
Source: Nat Commun. 2022 Jan 20;13:415. doi: 10.1038/s41467-022-28022-3 (PMC8776946; doi:10.1038/s41467-022-28022-3)
Supplement: Supplementary file 2 — Peer Review File [file 41467_2022_28022_MOESM2_ESM.pdf]

nature portfolio

Peer Review File

Selective Photoinduced Charge Separation in Perylenediimide-Pillar[5]arene Rotaxanes

Open Access This file is licensed under a Creative Commons Attribution 4.0 International License, which permits use, sharing, adaptation, distribution and reproduction in any medium or format, as long as you give appropriate credit to the original author(s) and the source, provide a link to the Creative Commons license, and indicate if changes were made. In the cases where the authors are anonymous, such as is the case for the reports of anonymous peer reviewers, author attribution should be to 'Anonymous Referee' followed by a clear attribution to the source work. The images or other third party material in this file are included in the article's Creative Commons license, unless indicated otherwise in a credit line to the material. If material is not included in the article's Creative Commons license and your intended use is not permitted by statutory regulation or exceeds the permitted use, you will need to obtain permission directly from the copyright holder. To view a copy of this license, visit http://creativecommons.org/licenses/by/4.0/.

Reviewers' Comments:

Reviewer #1:
Remarks to the Author:
This is a very fine piece of work, and the quality of the presentation is excellent, way above average, except for one or two minor points made below. I am tempted to use the term elegant for the conception and execution of this project, which places it near the top of recent contributions in the field of photoinduced charge separation. I had not previously encountered rotaxanes containing electron donor and acceptor moieties similar to those studied here except of course for PDI. Publication in Nature Communications is strongly recommended after a few very minor changes.
I think the structures of the target rotaxanes in Scheme 1 are too small. I like the way the Scheme is laid out, but it is difficult for me to see the details of the final structures in connection with the spectral observations.
I have never encountered the term "pillar[5]arene. Is that a well known structure designation or does it need explanation?
On page 3, why use the word "some". It seems unnecessary to me.
Page 4,line 7: What is "planar chirality"?
Page 4, last line: should be "or"
Page 7, middle: doesn't "hyperfine" need a subject noun?
Same page, last line: Shouldn't it be "both rotaxanes"?
Conclusion, last sentence: I don't know what they mean by "intramolecular symmetry". I think a much stronger concluding sentence is warranted.

Reviewer #2:
Remarks to the Author:
The authors report two photoactive rotaxanes. The structures were characterized by electron diffraction studies, and the authors state that this is a first for a mechanically interlocked molecule. This is followed by rather routine characterization studies such, as CV, electronic absorption, time resolved spectroscopy, FTIR, epr, which led authors to conclude that two different photoinduced charge separation processes take place with these rotaxanes. The data acquired are all expected behaviors for rotaxanes composed of photoactive units of complementary electron densities. The manuscript itself does not present a novel concept or idea, and much better suited for a specialized physical chemistry journal.

I suggest rejection.

Reviewer #3:
Remarks to the Author:
The communication submitted by Pearce and coworkers describes the interesting photoinduced charge transfer processes occurring in two rotaxanes of the [3]- and [4]rotaxane variety that are comprised of a perylenediimide-based axel and both pillar[5]arene and bis(1,5-naptho)-38-crown-10 macrocycles. The work is carried out to a very high degree of excellence and thoroughness. It is particularly noteworthy that rotaxanes are devilishly difficult to crystalize and obtain data suitable for structure elucidation using X-Ray crystallography. It is quite impressive, surprising, and exciting to see that the authors have succeeded in elucidating the solid state structure of the hetero [4]rotaxane. In fact, one of the most exciting components of this work is the solid state elucidation of this rotaxane and this reviewer believes that this aspect of the work is quite understated in both the abstract and in the main text. To this reviewer's knowledge, the authors are correct to state that this may very well be the first crystal structure of a hetero[n]rotaxane that has been solved. This is no small feat by any means and will certainly impact community in it of itself. Moreover, this reviewer has never read a manuscript which reports using the methodology that was used to solve the mechanically interlocked structure, using a combination of diffraction data from X-Ray crystallography and electron diffraction. The authors are right to also conclude that this another first presented in the manuscript in regards to solving the solid state

structure of a mechanically interlocked molecule. I would advise that the reviewers consider revising Figure 1a so that the solid state structure is a bit more impactful. Perhaps the addition of a space filling model or a transparent space filling model overlaid on the ball-and-stick representation would hit a bit harder?

The photophysical study is also very intriguing and complements quite well the structural information obtained from the solid state structure. While it is not completely surprising that there exists another charge transfer pathway for the [4]rotaxane versus the [3]rotaxane, what is surprising is just how one sided the switch is, in regards to engaging fully one pillar[5]arene as the donor in the [3]rotaxane and then solely engaging the 38-crown-10 macrocycle macrocycle as the donor in the [4]rotaxane. It is only in hindsight that such an observation appears intuitive but the reality is that one may have expected, as the authors pointed out, that charge transfer could have also occurred from the pillar[5]arene being that has a lower energy HOMO, but ultimately does not.

Another point of intrigue is the decomposition of the [4]rotaxane to the [3]rotaxane following bulk electrochemical oxidation. The authors reason that the [4]rotaxane loses the 38-crown-10 macrocycle in this process and indicate that MALDI-TOF analysis supports this claim. However, what is the mechanism of this process? Some clarification here should be much appreciated by the community. The authors comment that this result is in "contrast to the dethreading observed upon reduction of the previously reported PDI-BN38C10."

Overall, this is excellent work that should be considered for publication in Nature Communications following very minor revisions addressing the comments made above. It may also prove beneficial to the community if the authors would consider including proton NMR spectra in the supporting information to support the comments made on page 3 of the submission.

Reviewer #1 is very positive about the manuscript and we thank them for their kind comments. They ‘strongly’ recommend publication in Nature Communications after a few very minor changes.

The reviewer comments “I think the structures of the target rotaxanes in Scheme 1 are too small. I like the way the Scheme is laid out, but it is difficult for me to see the details of the final structures in connection with the spectral observations.”
We feel that the scheme follows accepted sizes for the structures. We have used the nature journals template and so we have not modified the scheme beyond the use of this template, although, to aid the reader we have emboldened the macrocyclic components of the rotaxanes to aid interpretation by the reader.

The reviewer says “I have never encountered the term "pillar[5]arene. Is that a well known structure designation or does it need explanation?”
Pillar[5]arene is a common term which, for the uninitiated can be found easily in the literature so we haven’t modified the text.

The reviewer also makes some detailed stylistic comments which we have addressed as follows:

- On page 3, why use the word "some". It seems unnecessary to me.
  Agreed, we have removed the word.
- Page 4,line 7: What is "planar chirality"?
  Planar chirality is found in molecules which would have a plane of symmetry if substituents did not break this symmetry. Thus, non-superimposable mirror images, and hence enantiomers, are observed. This is a well known term in the pillar[5]arene community.
- Page 4, last line: should be "or"
  We thank the reviewer for spotting this error and have corrected it.
- Page 7, middle: doesn't "hyperfine" need a subject noun?
  Yes, the reviewer is correct and we have corrected the text.
- Same page, last line: Shouldn't it be "both rotaxanes"?
  Thanks, we have corrected the text.
- Conclusion, last sentence: I don't know what they mean by "intramolecular symmetry". I think a much stronger concluding sentence is warranted.

We thank the reviewer for bringing this to our attention. We have simplified the text so it simply reads "molecular symmetry"

Reviewer #2 does not find the work exciting but offers no specific criticisms which can be addressed.

Reviewer #3 is very positive in their praise and we thank them for their warm comments as follows:

The communication submitted by Pearce and coworkers describes the interesting photoinduced charge transfer processes occurring in two rotaxanes of the [3]- and [4]rotaxane variety that are comprised of a perylenediimide-based axel and both pillar[5]arene and bis(1,5-naptho)-38-crown-10 macrocycles. The work is carried out to a very high degree of excellence and thoroughness. It is particularly noteworthy that rotaxanes are devilishly difficult to crystalize and obtain data suitable for structure elucidation using X-Ray crystallography. It is quite impressive, surprising, and exciting to see that the authors have succeeded in elucidating the solid state structure of the hetero [4]rotaxane. In fact, one of the most exciting components of this work is the solid state elucidation of this rotaxane and this reviewer believes that this aspect of the work is quite understated in both the abstract and in the main text. To this reviewer's knowledge, the authors are correct to state that this may very well be the first crystal structure of a hetero[n]rotaxane that has been solved. This is no small feat by any means and will certainly impact community in it of itself. Moreover, this reviewer has never read a manuscript which reports using the methodology that was used to solve the mechanically interlocked structure, using a combination of diffraction data from X-Ray crystallography and electron diffraction. The authors are right to also conclude that this another first presented in the manuscript in regards to solving the solid state structure of a mechanically interlocked molecule.

The reviewer says "I would advise that the reviewers consider revising Figure 1a so that the solid state structure is a bit more impactful. Perhaps the addition of a space filling model or a transparent space filling model overlaid on the ball-and-stick representation would hit a bit harder?"
As suggested, we have included an additional space filling model of the structure in Figure 1 to aid appreciation of the structure.

The reviewer continues "The photophysical study is also very intriguing and complements quite well the structural information obtained from the solid state structure. While it is not completely surprising that there exists another charge transfer pathway for the [4]rotaxane versus the [3]rotaxane, what is surprising is just how one sided the switch is, in regards to engaging fully one pillar[5]arene as the donor in the [3]rotaxane and then solely engaging the 38-crown-10 macrocycle macrocycle as the donor in the [4]rotaxane. It is only in hindsight that such an observation appears intuitive but the reality is that one may have expected, as the authors pointed out, that charge transfer could have also occurred from the pillar[5]arene being that has a lower energy HOMO, but ultimately does not.

Another point of intrigue is the decomposition of the [4]rotaxane to the [3]rotaxane

following bulk electrochemical oxidation. The authors reason that the [4]rotaxane loses the 38-crown-10 macrocycle in this process and indicate that MALDI-TOF analysis supports this claim. However, what is the mechanism of this process? Some clarification here should be much appreciated by the community. The authors comment that this result is in "contrast to the dethreading observed upon reduction of the previously reported PDI-BN38C10."

This is an interesting point and we thank the reviewer for raising it. Indeed, the process of dethreading is both intriguing and challenging. We have added the text in the main manuscript to address the point:

"Dethreading of the BN38C10 macrocycle, presumably driven by electrostatic repulsion between positively charged macrocyles, can only be achieved by the BN38C10 macrocycle passing over one of the P5A components. Inspection of the dimensions of the two macrocycles suggests that BN38C10 does indeed have an internal area that is large enough to pass over P5A and considering the additional flexibility of the crown ether, in comparison to the comparatively rigid nature of P5A, we propose that this process provides a pathway for dethreading. Indeed, ring-through-ring threading has been reported previously for rotaxanes comprising of crown ether macrocycles.[36]"

The new reference (ref 36) has also been added.

The reviewer continues "Overall, this is excellent work that should be considered for publication in Nature Communications following very minor revisions addressing the comments made above. It may also prove beneficial to the community if the authors would consider including proton NMR spectra in the supporting information to support the comments made on page 3 of the submission."

The NMR spectra have been included in the supporting information file as suggested.

We have uploaded the revised manuscript and supporting information, accompanied by the author checklist and associated documents. We thank the reviewers for their helpful comments and suggestions and we hope that the manuscript is now acceptable for *Nature Communications*,

Yours sincerely,

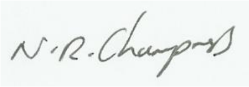

Professor Neil Champness
